# Removing seasonal confectionery from prominent store locations and purchasing behaviour within a major UK supermarket: Evaluation of a nonrandomised controlled intervention study

**DOI:** 10.1371/journal.pmed.1003951

**Published:** 2022-03-24

**Authors:** Carmen Piernas, Georgina Harmer, Susan A. Jebb

**Affiliations:** Nuffield Department of Primary Care Health Sciences, University of Oxford, Oxford, United Kingdom; University of Cambridge, UNITED KINGDOM

## Abstract

**Background:**

The proportion of energy from free sugars and saturated fat currently exceeds the UK-recommended intake across all age groups. Recognising the limits of reformulation programmes, the government in England has announced their intention to introduce legislation to restrict the promotion of foods high in free sugars, salt, and saturated fats in prominent store locations. Here, we evaluated a grocery store intervention to remove seasonal confectionery from prominent locations within a major UK supermarket.

**Methods and findings:**

A nonrandomised controlled intervention study with interrupted time series (ITS) analysis was used. Data were analysed from 34 intervention stores located in 2 London boroughs and 151 matched control stores located elsewhere in the UK owned by the same retailer. Stores were matched based on store size and overall sales during the previous year. Between 15 February 2019 and 3 April 2019 (before Easter), stores removed free-standing promotional display units of seasonal confectionery from prominent areas, although these products were available for purchase elsewhere in the store.

Store-level weekly sales (units, weight (g), and value (£)) of seasonal chocolate confectionery products were used in primary analyses, with data from 1 January 2018 to 24 November 2019. Secondary outcomes included total energy, fat, saturated fat, and sugars from all in-store purchases. Multivariable hierarchical models were used to investigate pre/post differences in weekly sales of confectionery in intervention versus control stores. ITS analyses were used to evaluate differences in level and trends after intervention implementation.

Over a preintervention baseline period (15 February 2018 to 3 April 2018), there were no significant differences in sales (units, weight, and value) of all chocolate confectionery between intervention versus control stores. After intervention implementation, there was an attenuation in the seasonal increase of confectionery sales (units) in intervention stores compared to control (+5% versus +18%; *P* < 0.001), with similar effects on weight (g) (+12% versus +31%; *P* < 0.001) and value (£) (−3% versus +10%; *P* < 0.001). ITS analyses generally showed statistically significant differences in the level at the point of intervention (*P* ranges 0.010 to 0.067) but also in the trend afterwards (*P* ranges 0.024 to 0.053), indicating that the initial difference between intervention and control stores reduced over time. There was a significant difference in level change in total energy sold, adjusted for the total weight of food and drink (kcal/g, *P* = 0.002), and total fat (fat/g) (*P* = 0.023), but no significant changes in saturated fat or sugars from total sales in ITS models. There was no evidence that the main results varied across store deprivation index. The limitations of this study include the lack of randomisation, residual confounding from unmeasured variables, absolute differences in trends and sales between intervention versus control stores, and no independent measures of intervention fidelity.

**Conclusions:**

Removal of chocolate confectionery from prominent locations was associated with reduced purchases of these products, of sufficient magnitude to observe a reduction in the energy content of total food purchases. These results from a “real-world” intervention provide promising evidence that the proposed legislation in England to restrict promotions of less healthy items in prominent locations may help reduce overconsumption.

**Trial registration:**

https://osf.io/br96f/.

## Abstract 2

Carmen Piernas and team evaluate purchasing behaviour associated with a grocery store intervention to remove seasonal confectionery from prominent locations within a major UK supermarket.

## Author summary

**Why was the study done?:**

- The prevalence of obesity in the UK is continuing to increase especially in the most deprived areas.
- The proportion of energy from free sugars and saturated fat currently exceeds the UK-recommended intake across all age groups. Despite ambitious sugar reduction targets set by Public Health England, there has been little change in the confectionery category.
- Recognising the limits of reformulation programmes, the Government in England has announced their intention to introduce legislation to restrict the promotion of foods high in free sugars, salt, and saturated fats in prominent locations in grocery stores.

**What did the researchers find?:**

- We partnered with a large UK food retailer to evaluate an intervention to remove seasonal chocolate confectionery from prominent areas of the store, specifically end-of-aisles and entrance areas, over 7 weeks before the Easter period.
- The intervention showed a significant attenuation of the seasonal increase in confectionery sales in intervention stores compared to control stores, with an absolute difference of approximately 127 units (approximately 21 kg) of confectionery per store per week.
- During the intervention period, there were significant reductions in total energy and fat from all food-related purchases in the stores implementing the intervention, but no significant changes in saturated fat or total sugars.
- There was no evidence that the main results varied according to the store deprivation index.

**What do these findings mean?:**

- The Government in England has recently signalled its intention to bring forward legislation to restrict promotions of less healthy items in prominent locations in grocery stores, to help reduce overconsumption.
- This study provides new evidence that the government proposals to restrict foods high in fat, sugar, and salt from prominent locations in stores could lead to measurable reductions in total energy (calories) from purchases.

## Introduction

Obesity is a global public health issue [1]. Currently in the UK, 26% of men, 29% of women, and 20% of children aged 10 to 11 years old have obesity, with significantly higher prevalence in the most deprived areas [2]. Despite years of health promotion to encourage and motivate individuals to choose a healthier diet, the proportion of energy coming from free sugars and saturated fat in the UK continues to exceed the recommended levels across all age groups with especially high intakes of free sugars among children [3]. A nutritionally poor diet increases the burden of major chronic diseases, including diabetes and cardiovascular disease, principally through increases in body weight as well as blood cholesterol, blood pressure, and insulin resistance [4–8].

Interventions to change food purchasing habits at the point of choice offer an upstream opportunity to change behaviour rather than relying on influencing consumption at the moment of eating. The World Health Organisation and other groups have advocated for the implementation of health-related taxes [9,10], but the acceptability of these interventions is relatively low [11,12]. Governments are increasingly interested in policy interventions to change supermarket environments because of the potential of these interventions to achieve population-level change in dietary habits and with higher public acceptability ratings [13–16].

Although sugar-sweetened beverages (SSBs) have been a major focus of policy actions [17], confectionery, together with cakes and biscuits, make a greater contribution to free sugars, saturated fat, and total energy intakes in the UK population [3]. Sales of chocolate and sugar confectionery in the UK have increased by 16.3% and 7.3%, respectively, in the last 5 years [18], with the highest purchases in the least affluent households [19]. Despite ambitious sugar reduction targets set by Public Health England there has been almost no change in the period 2015 to 2019, with sugar in sweet and chocolate confectionery reducing by just 0.1% and 0.4%, respectively, against the 20% reduction target [18]. This is perhaps because of the challenges of reformulation for this food category, especially compared to SSBs with their potential to use nonnutritive sweeteners. Instead, any reductions in calories, sugar, and saturated fat from confectionery are likely to depend upon reducing the volume of confectionery consumed. All food is rewarding, but the combination of high energy, fat, and sugar in confectionery is associated with strong and reinforcing biological signals [20]. There are also powerful social norms and cultural traditions that foster the notion of confectionery as a treat, and, thus, people tend to discount the long-term harms in favour of the short-term reward [21]. Few people consider confectionery to be a healthy food, and education alone is unlikely to be successful in reversing these powerful biological and societal drivers of consumption.

The 2020 obesity plan in England proposed new legislation to restrict volume- and location-based promotions on unhealthier products (i.e., those high in fat, salt, and sugar), as well as the placement of these products in prominent locations within supermarkets [16]. Placement and price promotions, together with availability, have been identified in previous systematic reviews of in-store interventions as potentially effective strategies to influence food purchasing behaviours [22–32]. According to the typology of interventions in proximal physical microenvironments (TIPPME) framework, availability and placement strategies work by increasing the range, variety, number, as well as visibility and accessibility of products, and this can stimulate purchases [33].

Most reviews have generally highlighted the lack of high-quality evidence in real supermarkets, especially for interventions that disincentive purchases of less healthy options [23,24,26,32]. In collaboration with the Consumer Goods Forum (CGF), a global membership body of 400 major consumer goods retailers and manufacturers, and with agreements enabling access to sales data from a major UK supermarket, we conducted an independent evaluation of an intervention, designed and implemented by a national food retailer, to remove seasonal chocolate confectionery from prominent store locations before the Easter period.

## Methods

This study is reported as per the Strengthening the Reporting of Observational Studies in Epidemiology (STROBE) guideline (S1 Checklist).

### Study design and data source

Data from a major UK retailer (comprising 27.7% of the UK grocery market share in January 2019) were used in this project. The study was completely developed and implemented by the retail partner, so we followed the methods suggested for the monitoring and evaluation of natural experiments [34], and a nonrandomised controlled design was used. The study was implemented in 34 stores (hereafter referred to as intervention stores), with a matched sample of 151 unique control stores. Data on store-level weekly sales of seasonal chocolate confectionery (units, weight [g], and value [£] of each eligible product within the category) were obtained for both intervention and control stores, spanning dates from 1 January 2018 to 24 November 2019 (with 4 weeks missing from 26 November 2018 to 30 December 2018 from all stores), which comprised a total of 17,380 aggregated store-week data points (see flowchart of store data in Fig A in S1 Appendix). Data from nutrients in all food-related sales, including total energy, sugar, saturated fat, and total fat, were available from 1 January 2019 to 24 November 2019.

By using aggregated weekly sales data, this study was exempt from ethical review and approval. A preregistered protocol (https://osf.io/br96f/) was completed and fully available from 22 July 2020 before obtaining data for analysis.

### Store selection and matching

Retail partner’s finance and data teams used proprietary analytics to select intervention and control stores for this study with no input from the research team. Based on each retailer’s operational considerations and with input from the CGF and the project partner, Impact on Urban Health, intervention stores were selected within London boroughs (Lambeth and Southwark, UK). The sample of intervention stores were located in neighbourhoods covering a range of socioeconomic deprivation strata based on the 2019 English Index of Multiple Deprivation (IMD) income domain, the official measure of relative deprivation in small areas (Lower-layer Super Output Areas) across England [35]. Selected intervention stores were all small supermarkets according to a retail food outlet categorisation system previously defined, which includes stores with 1 to 4 manned cash registers [36,37]. Control stores were selected across each retailer own stores, with store size and overall sales performance over the previous year used as the criteria for matching stores.

### Intervention

The intervention aimed to reduce the extra availability of seasonal chocolate confectionery by removing free-standing promotional display units from prominent areas, for example, store entrance, as well as by substituting seasonal confectionery located in end of aisles with other products. A total of 178 uniquely barcoded products were removed from display units or end of aisles, but all these products were still available for purchase elsewhere in the store (although many of these products were seasonal and only available during a short period of time). The intervention was implemented in the run-up to the Easter period, for approximately 7 weeks (15 February 2019 to 3 April 2019) with a phased implementation: 17% of eligible products were removed from 15 February 2019; 53% more were removed from 13 March 2019; and the remaining 30% were removed from 18 March 2019.

### Outcome measures

Primary outcome measures included store-level weekly sales data (units, weight, and value) for the whole category of seasonal chocolate confectionery. Secondary outcome measures included nutrient data (i.e., total energy, sugar, fibre, saturated fat, and total fat) from all food-related sales.

### Store characteristics

Store characteristics relating to the customer population included the English IMD and ethnicity. The store postcode was matched to the IMD income domain, the official measure of relative deprivation in small areas (Lower-layer Super Output Areas) across England [38], which was used as a proxy for the socioeconomic status (SES) of the customer population. The store sample covered neighbourhoods from deciles 1 to 10; regrouped into IMD 1 to 3 (most deprived), 4 to 6 (mid), and 7 to 10 (least deprived). Ethnicity of the store customer population was classified by the retailer using internal proprietary systems and grouped as predominantly white versus other ethnicities.

### Statistical analysis

Power analyses were not conducted, and the retailer chose the number of stores to roll out the interventions.

Descriptive analyses were used to investigate differences in store demographic characteristics between intervention and control stores using χ^2^ tests. We used data over the year prior to intervention (2018) to define preintervention baseline period (15 February 2018 to 3 April 2018), which matched as much as possible the intervention period. We tested differences in weekly sales of target products over the 2018 baseline periods between intervention and control stores using Student *t* tests.

The following prespecified statistical models were used for the primary and secondary outcome analyses, using consistent methods for intervention evaluation [39]:

- Hierarchical models (negative binomial for unit sales; or linear mixed models for weight and value of purchases) were used with a fixed effect adjustment for store demographic characteristics and average weekly sales over the baseline preintervention period. This model was used to investigate differences in weekly sales of target products in intervention versus control stores over the time period while the intervention was active compared to the preintervention baseline period (2018) [40].
- Interrupted time series (ITS) analyses and corresponding plots with fitted linear trends were computed using all available data before and after the intervention for intervention and control stores [41]. To assess whether differences visible in the graphs were statistically significant between intervention and control stores and to account for any preintervention differences between groups in the outcome variable, we used a difference-in-difference approach, calculating the mean difference in weekly sales between intervention and control stores, and testing whether this time series of differences changed after versus before intervention using a linear regression model. We used a Chow-type test for level and trend changes after intervention implementation, and Newey–West standard errors with lag 4 to allow for autocorrelation in the time series. Since intervention implementation was phased, we conducted one model where intervention started on the week of 15 February 2019 when 17% of products were removed, and a second model where intervention started on the week of 13 March when 53% more products were removed.

Analyses were conducted using all intervention and control stores with all available data. A prespecified exploratory subgroup analysis on unit sales was performed by store IMD groups (IMD 1 to 3 high deprivation versus IMD 4 to 10 middle/low deprivation), and likelihood ratio tests were used to test the significance of interactions. Stata version 16 was used for all statistical tests with a 5% significance level.

## Results

### Differences in store characteristics

A total of 185 stores were analysed, with all the intervention stores located in areas of medium or high deprivation, which is representative of the population of Lambeth and Southwark (London, UK). The control group had 28% of stores in areas of low deprivation (Table 1). There were significant differences in IMD scores (*P* < 0.001) but not in ethnicity between intervention and control stores.

**Table 1:** Store demographic characteristics.

	Total stores		Intervention stores		Control stores		χ^2^ test
	*N =* 185	%	*n =* 34	%	*n* = 151	%	*P* value
***IMD score groups***							
IMD 1–3 (most deprived)	52	28	18	53	34	23	<0.001
IMD 4–6	91	49	16	47	75	49	
IMD 7–10 (least deprived)	42	23	0	0	42	28	
***Ethnicity***							
Predominantly white	49	27	7	21	42	28	0.388
Other ethnicities	136	73	27	79	109	72	

IMD, Index of Multiple Deprivation.

### Primary analysis—Sales of confectionery

Over a preintervention baseline period (15 February 2018 to 3 April 2018), there were no significant differences in sales (units, weight, and value) of all chocolate confectionery between intervention versus control stores (Table 2).

**Table 2:** Average weekly sales of confectionery in intervention vs. control stores and comparison of changes before/after intervention between intervention vs. control stores.

	Baseline period 15 Feb– 3 April 2018			Intervention period 15 Feb– 3 April 2019					*Comparison intervention vs*. *control stores*			
	Average sales			Average sales		Absolute difference vs. baseline period		% Change				
**Units/store/week**	**Mean**	**SD**	***P* value** ^*^	**Mean**	**SD**	**Mean**	**SD**		**IRR** ^**†**^	**95% CI**		***P* value**
Intervention stores	894.2	202.9	0.070	938.1	304.8	43.9	162.8	5%	0.861	0.808	0.918	<0.001
Control stores	966.7	267.9		1,137.6	368.6	170.8	195.8	18%	0.864	0.809	0.922	<0.001
**Weight (g)/store/week**	**Mean**	**SD**	***P* value** ^*^	**Mean**	**SD**	**Mean**	**SD**		**β** ^**†**^	**95% CI**		***P* value**
Intervention stores	97,172.2	25,332.2	0.074	108,650.2	36,058.1	11,478.1	17,434.8	12%	−20,416.5	−28,373.6	−12,459.3	<0.001
Control stores	105,554.9	31,309.0		137,827.8	45,876.4	32,272.9	21,601.4	31%	−21,790.1	−30,228.9	−13,351.3	<0.001
**Value (£)/store/week**	**Mean**	**SD**	***P* value** ^*^	**Mean**	**SD**	**Mean**	**SD**		**β** ^**†**^	**95% CI**		***P* value**
Intervention stores	1,096.4	287.7	0.058	1,067.7	351.1	−28.7	144.4	−3%	−164.1	−227.2	−101.1	<0.001
Control stores	1,198.9	352.6		1,323.9	415.3	125.0	175.4	10%	−176.7	−241.4	−112.1	<0.001

*Student *t* tests comparing average sales over the baseline period between intervention vs. control stores.

^**†**^IRRs from hierarchical negative binomial models (used in the models of unit sales), minimally adjusted for average sales per week over the 2018 period (top row) or fully adjusted (bottom row) with fixed effect adjustment for store ethnicity, IMD, and average sales per week over the 2018 period; Beta (β) coefficients from hierarchical normal mixed models (used in the models of gr and £ sales), minimally adjusted for average sales per week over the 2018 period (top row) or fully adjusted (bottom row) with fixed effect adjustment for store ethnicity, IMD, and average sales per week over the 2018 period.

IMD, Index of Multiple Deprivation; IRR, incidence rate ratio.

After intervention implementation, there was an attenuation in the seasonal increase of confectionery sales (units) in intervention stores compared to control (+5% versus +18%; *P* < 0.001), with similar effects on weight (+12% versus +31%; *P* < 0.001) between 15 February 2019 to 3 April 2019. However, there was a decrease in value sales in intervention compared to control stores over the same time period (−3% versus +10%; *P* < 0.001) (Table 2). There were absolute differences in confectionery sales of approximately 127 units per store per week (+43.9 units in intervention stores versus +170.8 units in control stores over the intervention period compared to the baseline period) or 21 kg (11.5 kg in intervention stores versus 32.3 kg in control stores) (Table 2).

**Fig 1:**
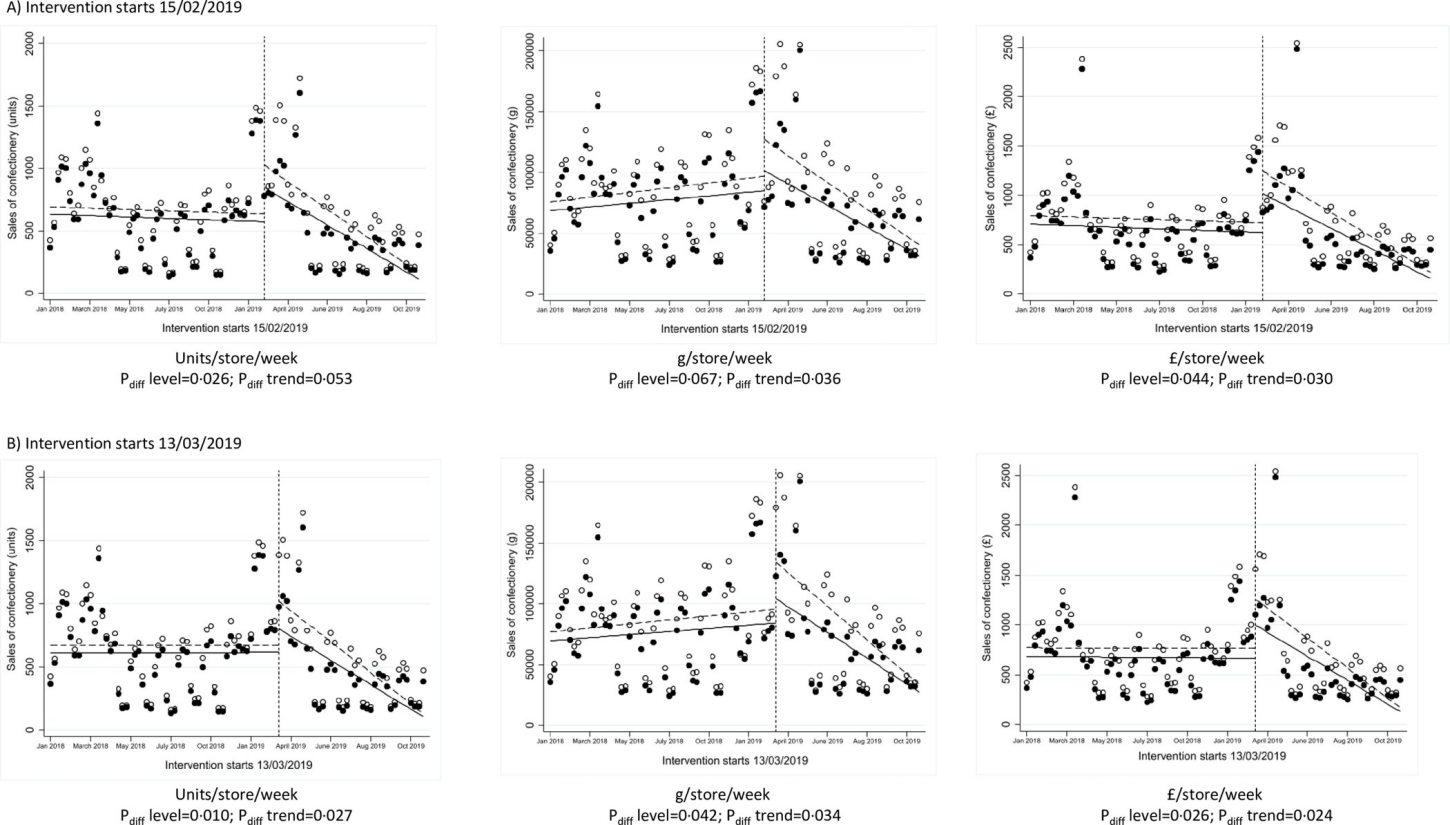
ITS analysis showing level and trend changes in weekly sales of confectionery. *Solid dots (observed) and lines (modelled) represent intervention stores; white dots (observed) and dotted lines (modelled) represent control stores. ITS, interrupted time series.

ITS analyses were conducted using 2 different time points for intervention implementation, firstly on 15 February 2019 when 17% of products were removed and secondly on 13 March 2019 when 53% more of products were removed. The trends before intervention implementation were generally consistent between intervention and control stores (Fig 1, Table A in S1 Appendix). After intervention implementation on 15 February 2019, there was a statistically significant difference in the level of weekly sales (Fig 1; *P*_*diff*_ *level =* 0.026 in units/store/week and *P*_*diff*_ *level =* 0.044 in £/store/week). There were stronger differences in level after intervention intensification on 13 March 2019 (Fig 1; *P*_*diff*_ *level =* 0.010 in units/store/week, *P*_*diff*_ *level =* 0.042 in g/store/week and *P*_*diff*_ *level =* 0.026 in £/store/week). There were generally significant differences in the downward trends afterwards, indicating that the initial difference between intervention and control stores reduced over time.

### Secondary analyses—Changes in energy and nutrients

**Fig 2:**
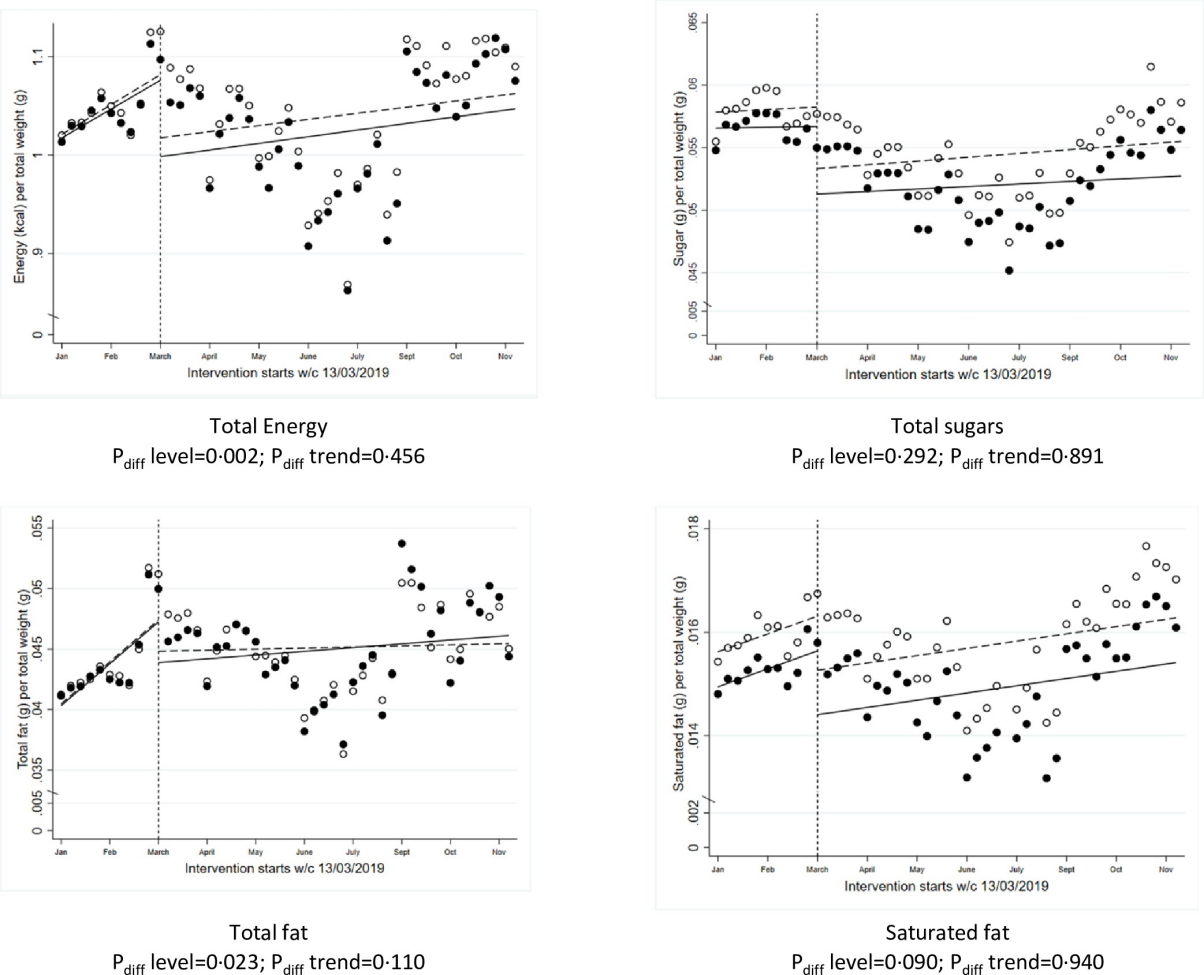
ITS analysis showing level and trend changes in calories and nutrients from all sales (averages per store/week) during the implementation of the chocolate confectionery availability study from 1 January 2019 to 24 November 2019. *Solid dots (observed) and lines (modelled) represent intervention stores; white dots (observed) and dotted lines (modelled) represent control stores. ITS, interrupted time series.

Data on total energy (calories), total sugars, total fat, and saturated fat from all food-related sales for the 2019 year were used in ITS models to evaluate the impact of the intervention on the overall healthiness of grocery shopping (Fig 2). There was a significant level change in total energy sold, adjusted for the total weight of food and drink (kcal/g, *P*_*diff*_ *level* = 0.002), and total fat (fat/g, *P*_*diff*_ *level* = 0.023), but no significant changes in saturated fat or sugars from total sales in ITS models. There were no significant differences in the trends afterwards for any of the nutrients studied.

### Differences by store deprivation

There was no evidence that the results varied across store IMD group for any of the metrics reported, with significant reductions in units, weight, and value in intervention stores located in high deprivation areas as well as those in mid or lower deprivation areas, compared to control stores (Table 3).

**Table 3:** Comparison of changes in sales of confectionery before/after intervention between intervention vs. control stores, across store IMD groups.

	*Comparison intervention vs*. *control stores*				
**Units/store/week**	**IRR** ^*^	**95% CI**		***P* value**	**P interaction** ^**†**^
IMD 1–3 high deprivation	0.90	0.83	0.98	0.010	0.795
IMD 4–10 medium/low deprivation	0.86	0.79	0.95	0.001	
**Weight (g)/store/week**	**β** ^*^	**95% CI**		***P* value**	**P interaction** ^**†**^
IMD 1–3 high deprivation	−18,571.0	−29,205.5	−7,936.4	0.001	0.775
IMD 4–10 medium/low deprivation	−20,935.8	−32,566.5	−9,305.1	<0.001	
**Value (£)/store/week**	**β** ^*^	**95% CI**		***P* value**	**P interaction** ^**†**^
IMD 1–3 high deprivation	−103.0	−175.3	−30.8	0.005	0.156
IMD 4–10 medium/low deprivation	−204.5	−298.2	−110.7	<0.001	

*IRRs from hierarchical negative binomial models (used in the models of unit sales), with fixed effect adjustment for store ethnicity, IMD, and average sales per week over the 2018 period; Beta (β) coefficients from hierarchical normal mixed models (used in the models of gr and £ sales), with fixed effect adjustment for store ethnicity, IMD, and average sales per week over the 2018 period.

**†**P interaction from likelihood ratio tests.

IMD, Index of Multiple Deprivation; IRR, incidence rate ratio.

## Discussion

This intervention to remove chocolate confectionery in prominent areas of the store showed a significant attenuation in the seasonal increase of confectionery sales (units) in intervention stores compared to control (+5% versus +18%; *P* < 0.001). Similar results were observed with weight (g) of confectionery (+12% versus +31%; *P* < 0.001) and value (£) (−3% versus +10%; *P* < 0.001), with an overall absolute difference between intervention and control stores of approximately 127 units (approximately 21 kg) of confectionery per store per week. In parallel, we observed significant reductions in total energy and fat from all food-related sales, but no significant differences in saturated fat or total sugars. There was no evidence that the intervention results varied according to the level of deprivation in the area in which the store was sited.

Systematic reviews of grocery store interventions have reported that positioning products in prominent locations, such as near checkouts or the end of an aisle, increases visibility of products and stimulates purchases [22–25]. A previous natural experiment across 12 stores in the US found that prominent positioning of sweet snacks at the end-of-aisles had a greater effect on sales of less healthier options compared to prominent positioning of the healthier ones and copositioning of both significantly increased sales of the unhealthier options only [42]. An observational study using sales from a UK grocery store estimated a 52% higher weekly volume sales of carbonated drinks when these were displayed in end of aisles [43]. But there is an important gap in the evidence with regard to interventions that can reduce the prominent positioning of unhealthy food, as most of the literature has focused on selling more healthy foods. A recent cluster RCT in Australian supermarkets tested a complex intervention to limit in-store promotional and marketing activities targeting high-fat/high-sugar products, including removal of price promotions, signage, and removal of products from prominent areas, and showed significant reductions in total sugars without affecting supermarket profit [44]. By just removing confectionery from prominent store locations, our study showed a significant attenuation in pre-Easter sales (units, weight, and value) of confectionery.

In the context of the increasing gap in dietary inequalities and long-term health outcomes, it is also important to understand if supermarket interventions help reduce, or at least do not exacerbate, dietary inequalities. It has been postulated that, compared to individual-level interventions, population-level approaches that trigger automatic (rather than conscious) behavioural responses [45,46] may be less likely to increase health inequalities. But evidence from systematic reviews is limited and most studies testing positioning interventions have not specifically looked at differential effects across sociodemographic groups [23,32]. Our results showed no evidence of differences in intervention results, and the intervention appeared to work equally well regardless of the area deprivation score of the store.

The 2020 obesity plan in England has laid out plans to introduce legislation to restrict the promotion of foods high in fat, sugar, or salt (HFSS), by restricting volume-based promotions such as “Buy One Get One Free” as well as restrictions to placement in prominent locations intended to encourage purchasing, both online and in physical stores [16]. Our results provide direct evidence on the reduced availability of chocolate confectionery in prominent locations, which will be of interest to policymakers and could help shape effective policies for confectionery and potentially other items.

This research was made possible through collaboration with food retailers, facilitated by an established industry programme led by the CGF to encourage healthier and more sustainable retail practices. This evaluation provides proof-of-concept that it is possible to establish these collaborations and has led to useful lessons for future collaborations, especially in relation to contractual agreements, and the design of larger and more definitive intervention studies. For example, the duration of intervention was limited here because of the seasonal nature of the products, but future studies of positioning interventions should aim to try implement the intervention for a longer time period. This is important since the trends from ITS models after implementation suggested that the effect of the intervention may be short-lived, though this may be related to the seasonal nature of the products targeted (i.e., a large proportion of the products targeted in the intervention are not available the rest of the year). Our analysis of the nutrient content of the total sales showed some evidence of no compensatory behaviours at least within the same retailer, although other research would need to investigate if customers are purchasing confectionery in different stores where no restrictions are imposed. Future research should also seek to analyse changes in purchases at a household rather than store level using data from customer loyalty cards rather than store-level sales to better study any potential impact of interventions on health inequalities [47,48].

A major strength of this study is the use of a large dataset of objectively collected sales data, which is generalizable to all customers of the participating stores over the studied time period. Data were available over an extended time period and drawn from an intervention conducted in real supermarkets, which can provide important insights to inform population-level interventions to encourage healthier food purchasing. However, this “real-world” intervention study presents analytical challenges. Adjustment for confounding and other sources of heterogeneity was approached in several ways. Firstly, control stores were matched to intervention stores, with more than one control store per intervention store. Matching was done using store demographic factors and overall sales over the previous periods, which, in this case, resulted in nonsignificant differences in baseline sales between intervention and control stores. However, there were significant differences by IMD due to the fact that stores in less deprived areas were underrepresented in the intervention group, though we adjusted for deprivation in the models. The difference-in-difference approach used in ITS models also helps to remove the effect of any small absolute differences in sales between the intervention and control stores. Finally, with access to extended periods of time (2018 and 2019), we were able to use the 2018 period as a control in the models. Other limitations to note include the lack of randomisation, residual confounding from unmeasured variables, and absolute differences in trends and sales between intervention versus control stores. There could have been other interventions in stores running alongside the one evaluated here, which could have influenced the observed effects, but the use of control stores could potentially adjust for this. In addition, we have no independent measures of intervention fidelity and we had to rely on the retailer implementation plans, which means suboptimal implementation may have diminished the apparent effects of this intervention. The intervention was selected, developed, and implemented by the retailer, without the direct involvement of the research group. It is not possible to know the extent to which this was influenced by behavioural theory, prior commercial insights, or awareness of government thinking, though it is probable that all contributed to greater or lesser extent. Finally, there was limited data on store characteristics, and the retailer provided only restricted data on the ethnicity of the customer population. The very broad categorisation of ethnicity is unlikely to have removed all of the confounding related to ethnicity in our results, although there were no significant differences in the distribution of ethnicity between intervention and control stores. Similarly, the IMD used as a measure of store deprivation may also be a very crude proxy for the SES status of the customer population, particularly when people drive to larger out-of-town supermarkets or for smaller stores located in city centres with a large proportion of nonlocal customers.

There is limited evidence for effective interventions to discourage food options that contribute the most energy, saturated fat, and free sugars [3], particularly confectionery, biscuits, or cakes. These results showed that removal of seasonal confectionery in prominent locations is a promising strategy to reduce unhealthy food purchasing behaviours, with changes in just one subcategory of foods of sufficient magnitude to observe reductions in the energy content of total food purchases. These results provide promising evidence that the proposed legislation in England to restrict promotions of less healthy items in prominent locations may help reduce overconsumption.

## Supporting information

S1 Checklist STROBE Checklist. (DOCX)

S1 Appendix Supplementary table and figure. **Fig A**. Flowchart of store data. **Table A**. Model-based estimates of the difference-in-difference interrupted time series (β coefficients (95% CI)) of mean baseline trend, post-implementation level change, and post-implementation trend change.
(DOCX)

## Abbreviations

CGF: Consumer Goods Forum
HFSS: high in fat, sugar, or salt
IMD: Index of Multiple Deprivation
ITS: interrupted time series
SES: socioeconomic status
SSB: sugar-sweetened beverage
TIPPME: typology of interventions in proximal physical microenvironments
