# Supplementary material for: Removing seasonal confectionery from prominent store locations and purchasing behaviour within a major UK supermarket: Evaluation of a nonrandomised controlled intervention study
Source: PLoS Med. 2022 Mar 24;19(3):e1003951. doi: 10.1371/journal.pmed.1003951 (PMC8946674; doi:10.1371/journal.pmed.1003951)
Supplement: S1 Appendix — Fig A. Flowchart of store data. Table A. Model-based estimates of the difference-in-difference interrupted time series (β coefficients (95% CI)) of mean baseline trend, post-implementation level change, and post-implementation trend change. (DOCX) [file pmed.1003951.s002.docx]

**Supplementary tables and figures**

**Fig A - Flowchart of store data**

Final sample size

N=17,380 store/weeks

Available outcome measures:

1) Sales of seasonal chocolate confectionery (units, weight [g] and cost [£] from 1^st^ January 2018 to 24^th^ November 2019

2) Nutrients in all food-related sales: total energy, sugar, saturated fat and total fat from 1^st^ January 2019 to 24^th^ November 2019

Control stores

N=151

(14,798 store/weeks)

Intervention stores

N=34

(3,332 store/weeks)

Missing data

N=750 store/weeks from 26^th^ November 2018 to 30^th^ December 2018

**Table A – Model based estimates of the difference-in-difference interrupted time series (β coefficients (95%CI)) of mean baseline trend, post-implementation level change and post-implementation trend change.**

| Confectionery sales | Differences in  baseline trend | | | Difference in  post-implementation  level change | | | Difference in  post-implementation  trend change | | | |
| --- | --- | --- | --- | --- | --- | --- | --- | --- | --- | --- |
| *Intervention starts*  *w/c 15/02/2019* | **Mean** | **95% CI** | | **Mean** | **95% CI** | | **Mean** | **95% CI** | | |
| Units/store/week | -0·1 | -0·8 | 0·6 | -118·9 | -223·5 | -14·3 | 3·5 | -0·1 | 7·0 |  |
| Weight (g)/store/week | -89·4 | -192·1 | 13·2 | -13768·0 | -28547·7 | 1011·7 | 513·2 | 33·6 | 992·8 |  |
| Value (£)/store/week | -0·3 | -1·1 | 0·5 | -124·7 | -246·2 | -3·2 | 4·5 | 0·5 | 8·5 |  |
| *Intervention starts*  *w/c 13/03/2019* | **Mean** | **95% CI** | | **Mean** | **95% CI** | | **Mean** | **95% CI** | | |
| Units/store/week | 0·0 | -0·6 | 0·6 | -157·5 | -277·1 | -38·0 | 5·3 | 0·6 | 9·9 |  |
| Weight (g)/store/week | -72·0 | -151·4 | 7·4 | -18559·3 | -36403·6 | -715·0 | 734·4 | 56·1 | 1412·6 |  |
| Value (£)/store/week | -0·2 | -0·9 | 0·4 | -159·9 | -299·8 | -20·0 | 6·3 | 0·9 | 11·8 |  |
|  |  |  |  |  |  |  |  |  |  |  |
| Nutrients in sales | **Differences in**  **baseline trend** | | | **Difference in**  **post-implementation**  **level change** | | | **Difference in**  **post-implementation**  **trend change** | | | |
| *Intervention starts*  *w/c 13/03/2019* | **Mean** | **95% CI** | | **Mean** | **95% CI** | | **Mean** | **95% CI** | | |
| Total energy (kcal) per g | -0·000218 | -0·000999 | 0·000562 | -0·013505 | -0·022533 | -0·004477 | 0·000322 | -0·000689 | 0·001332 |  |
| Total sugars (g) per g | -0·000027 | -0·000133 | 0·000079 | -0·000479 | -0·001377 | 0·000419 | 0·000007 | -0·000098 | 0·000112 |  |
| Total fat (g) per g | -0·000001 | -0·000048 | 0·000046 | -0·000772 | -0·001481 | -0·000063 | 0·000046 | -0·000021 | 0·000112 |  |
| Saturated fat (g) per g | 0·000001 | -0·000020 | 0·000021 | -0·000188 | -0·000401 | 0·000024 | -0·000001 | -0·000021 | 0·000020 |  |
