# Supplementary material for: Removing seasonal confectionery from prominent store locations and purchasing behaviour within a major UK supermarket: Evaluation of a nonrandomised controlled intervention study
Source: PLoS Med. 2022 Mar 24;19(3):e1003951. doi: 10.1371/journal.pmed.1003951 (PMC8946674; doi:10.1371/journal.pmed.1003951)
Supplement: Attachment — Submitted filename: Response to reviewers CGF Conf trial 02 12 2021.docx [file pmed.1003951.s003.docx]

| Requests from the editors | | |
| --- | --- | --- |
| Comment | Response | Changes |
| Please add a final line to your abstract ‘Methods and findings’ which summarises the main limitations (it should begin ‘The limitations of this study include’ or similar). Given that the manuscript covers a variety of studies, it may be best to focus on the limitations relating to study design more generally as opposed to detailing limitations specific to any one of the experiments presented. | We have added this information | Abstract:  *“The limitations of this study are inherent to natural experiments and include the lack of randomisation, residual confounding from unmeasured variables, absolute differences in trends and sales between intervention vs control stores and no independent measures of intervention fidelity.”* |
| Please include line numbering in the margins throughout. | We have added line numbering |  |
| Citations should be in square brackets and occur before punctuation. | We have corrected this |  |
| As reflected in the reviewers’ reports, please provide further clarification as to the definition/classification of ethnicity in your studies and consider discussing it as a limitation. | We have clarified this in the methods and limitation sections. Unfortunately the information provided by the retailer on consumer ethnicity is considered sensitive and therefore the level of detail they provided to us was limited. | In Methods:  “Ethnicity *of the store customer population was classified by the retailer using internal proprietary systems, and grouped as predominantly white vs other-ethnicities*.”  In Discussion:  *“Finally, there was limited data on store characteristics, and the retailer provided only restricted data on the ethnicity of the customer population. The very broad categorisation of ethnicity is unlikely to have removed all of the confounding related to ethnicity in our results, although there were no significant differences in the distribution of ethnicity between intervention and control stores.”* |
| The PDF conversion process appears to have introduced some minor formatting errors – an example can be found in line 1 of page 8 of 3910 and also line 3 of 3912 (a boxed question mark appears where it should not). Please check throughout for other errors. | We have corrected this |  |
| Please remove the funding information from the acknowledgements section (in the event of publication this will be published as metadata based on your responses to the submission form).  Please also remove the Contributors, Competing interests, and Data sharing sections from the main text for the same reason as above. | We have removed this |  |
| Please update your Data Availability Statement (in the submission form) to include your statement regarding availability of statistical code. Note that a study author cannot be the main contact for researchers wishing to obtain this code, it would be better if this can be shared in a public repository or handled by an alternative data management contact. Please see our policy here (<https://journals.plos.org/plosmedicine/s/data-availability>) and get in touch if you have any questions. | We have updated this |  |
| Please remove the Ethical approval statement from the end of the main text as this is already stated in the methods section. | We have removed this |  |
| In your references, please remove all formatting (italics) and only use et al. after listing the first six authors. Please also ensure you have provided the data accessed for any URLs provided. Our guidelines can be found here: <https://journals.plos.org/plosmedicine/s/submission-guidelines#loc-references> | We have formatted the references accordingly |  |
| Please ensure you define all abbreviations used in your tables (e.g. IMD, IRR). | We have defined all abbreviations used in tables |  |
| Thank you for providing a STROBE checklist. Please update the checklist to reflect the combined manuscript and please use section names and paragraph numbers rather than page numbers (these are likely to change during the revision process). | We have uploaded a new STROBE checklist | TBD |
| Please reference the checklist in your methods ("This study is reported as per the Strengthening the Reporting of Observational Studies in Epidemiology (STROBE) guideline (S1 Checklist)." | We have added this to the methods section |  |
| When reporting p-values, please use P<0.001 rather than P<0.0001 (please also check your figures and tables to ensure P<0.001 is used consistently). | We have corrected this |  |
|  |  |  |
| Comments from the reviewers: | | |
| Reviewer #1 | Response | Changes |
| This is a well-conducted non-randomised controlled trial testing removal of seasonal confectionery from prominent store locations in a major UK supermarket. The study design, sample size, datasets, statistical methods and analyses, and presentation (tables and figures) and interpretation of the results are mostly adequate and of a good standard. The key issue in the non-randomised controlled trial setting is to control the potential confounding factors and minimise the biases which the authors have done well by adequate store selection and matching, and also adjusting in the analyses for store demographic characteristics such as deprivation score IMD and ethnicity and average weekly sales over the baseline pre-intervention period. However, there are still a few relatively minor issues needing attention. | We thank the reviewer for their positive comments and useful suggestions |  |
| 1) Adjustment for ethnicity. It's well known that London boroughs of Lambeth and Southwark have an urban multi-ethnic population with nearly half of population from ethnic minorities background, which is very different from other parts of the UK. In the paper, ethnicity is classified as predominantly white vs other ethnicities, which is a bit simplistic and non-specific. It is likely to have residual confounding in ethnicity even after such adjustment in the paper. It would be good if the authors can discuss this as a limitation in the paper. | We agree with the reviewer that the issue with ethnicity is particularly important for these London boroughs. Unfortunately there was limited information on the store characteristics and definitions of those characteristics that was provided to us by the retailer, as this is treated as sensitive information by the retail partner.  We tested for differences in store characteristics that could influence the study outcomes by comparing the distribution of the ethnic groups between intervention and control stores and found no significant differences, which should help reduce the possible bias introduced by this. In any case, we have included a brief mention to this in the methods and limitation section. | In Methods:  “Ethnicity *of the store customer population was classified by the retailer using internal proprietary systems, and grouped as predominantly white vs other-ethnicities*.”  In Discussion:  *“Finally, there was limited data on store characteristics, and the retailer provided only restricted data on the ethnicity of the customer population. The very broad categorisation of ethnicity is unlikely to have removed all of the confounding related to ethnicity in our results, although there were no significant differences in the distribution of ethnicity between intervention and control stores.”* |
| 2) In the abstract, it says "ITS analyses showed statistically significant differences in the level at the point of intervention (P<0·05 for confectionery sales)". As the authors are using 3 digits for P-values in the paper, it would be good to be consistent throughout. I am aware here P<0.05 refers to a range of p-values but can replace with, for example, P ranges from 0.024 to 0.064. Also, in the next sentence "but also in the trend afterwards, indicating that the initial difference between intervention and control stores reduced over time", p-value is needed to support the statement. | We have amended all p values to specify the detail to 3 decimal places. | In Abstract  “*ITS analyses generally showed statistically significant differences in the level at the point of intervention (P ranges 0·010 to 0·067) but also in the trend afterwards (P ranges 0·024 to 0·053), indicating that the initial difference between intervention and control stores reduced over time.”* |
| 3) In the first paragraph in the discussion, it says "This intervention to remove chocolate confectionery in prominent areas of the store attenuated the seasonal increase in confectionery sales, with an overall absolute difference between intervention and control stores of approximately 127 units (~21 kg) of confectionery per store per week". However, this 127 units just appeared out of blue and I had to figure it out myself. It's not mentioned in table or the results section at all, and it's not the primary outcome either. It would be good to give a message consistent with what's in the abstract which is on percentage differences in sales and etc. The absolute difference can be described as secondary but can't replace the primary outcome. | We have included this absolute difference in the results section with appropriate reference to the table. This is based on the primary outcome data showed in Table 2 and it’s meant to be an alternative way of presenting the results of the primary outcome which may be easier to understand and communicate to a wider audience. | In results:  “*There were absolute differences in confectionery sales of approximately 127 units per store per week (+43.9 units in intervention stores vs +170.8 units in control stores over the intervention period compared to the baseline period) or 21 kg (11.5 kg in intervention stores vs 32.3 kg in control stores) (Table 2).”* |
|  |  |  |
| Reviewer #2 | Response | Changes |
| I was asked to review these papers together. Given their similarity many of my comments apply equally to both and so I have provided a combined report. I've noted where comments refer particularly to one or the other paper and described these as the 'combined' or 'easter' papers. Overall, I wasn't convinced by the value of separating these papers. Whilst the combined paper is certainly complicated, I don't think that adding the easter analyses would make it appreciably more so. I think perhaps the only value of the distinct easter paper is that it speaks very specifically to current UK public health policy decisions. On the other hand, the specific high value placement message could be drawn out in post-publication dissemination and, arguably, UK public health policy needs to be thinking beyond just the current policy decisions on the table to some of the strategies presented in the combined paper. | We appreciate the reviewer views about the combination of results into a single paper. This is something we debated at length within the author group. As the reviewer notes, the analyses are incredibly complex and presenting results from multiple interventions makes it more so. We appreciate these trials share similarities, however they also have very important differences.  We decided to separate the intervention on confectionery from the rest because this intervention is the only one within the group that does not involve an implicit swap for a healthier option, this intervention aims to reduce the absolute amount of confectionery sold in stores. In contrast, the rest of the trials aimed to increase sales of healthier food options, through encouraging switches from less healthy to healthier options.  Secondly, the data we received to evaluate each trial was different for the confectionery trial. This is the only study for which we have data on the nutrient content of the overall shopping basket, and so we are able to examine potential compensatory effects. This is a much more robust analysis, hence singling it out. For the other six trials, we could only analyse the total sales of the specific category that was the target of the intervention. The remaining interventions are individually limited by the available data, but as reviewers note, by bringing them together we can comment on the relative impact of different behavioural mechanisms.  The confectionery trial is especially relevant for policy in light of the coming legislation and we believe the messages would get diluted if the manuscripts were combined. | In discussion with the editorial team it was agreed that the manuscripts would stay separate and changes suggested by the reviewer will be made to each where appropriate.  We have extracted the comments from the reviewer which relate specifically to this paper or to both. |
| These papers report on a rather disparate set of interventions in supermarkets aiming to improve the healthiness of purchases. I applaud the authors on the partnership they have developed with the respective retailers and agree that this represents a substantial achievement both in terms of interventions achieved and in data made available. Unfortunately I found the report (particularly in the combined paper) of, what is undoubtedly a complicated collection of work, rather hard to follow and a little underdeveloped in terms of both theoretical introduction and interpretation of findings. | We have revised the introduction section in the current paper (confectionery sales) | In Introduction:  “*Placement and price promotions, together with availability have been identified in previous systematic reviews of in-store interventions as potentially effective strategies to influence food purchasing behaviours. According to the typology of interventions in proximal physical micro-environments (TIPPME) framework, availability and placement strategies work by increasing the range, variety, number, as well as visibility and accessibility of products, and this can stimulate purchases”* |
| The nature of the partnership between the researchers and the retailers is a little unclear. Although there are a number of different definitions of natural experiments, and natural experimental evaluations, key is that the nature and implementation of the intervention is out with control of the researchers. It is not clear if this was the case here. If there is uncertainty in definitions, non-randomised controlled trials is an unambiguous description of what was done. | The trials were completely organised and implemented by the retailer without any involvement from the study team, therefore completely out of our control. We were invited as an evaluation partner. Hence naming them natural experiments. We have clarified the nature of the partnership. | In Methods:  “*The trial was completely developed and implemented by the retail partner, so we followed the methods employed in the monitoring and evaluation of a natural experiment, using a non-randomised controlled trial design*.” |
| I'd also like to understand more about why this particular group of interventions was selected for study. Was this informed by theory, pragmatic considerations or something else? A key limitation on how the findings can be interpreted is the possibility of an intervention modality by food category interaction - as different food categories were selected for each intervention modality, we can't tell if the effects reported are specific to the food category, the intervention modality, or the interaction of the two. I'd like to see more consideration given to this throughout.  I also thought there was a missed opportunity to raise the issue of industry versus government led public health initiatives in this paper. Again, it wasn't clear to me whether the decision to implement these interventions was driven by retailers, the industry body mentioned, the charity mentioned, or the researchers. Shedding some further light on this may help illuminate how such interventions might be implemented more consistently. | We thank the reviewer for this useful comment. As said above, both the choice of intervention modality and food category was entirely made by the retailers hence potentially not informed by theory. We have added a brief mention to this in the limitation section.  We have also clarified throughout that these were all retailer-led interventions. However it is very likely that the selection of interventions by retailers was influenced by real or perceived government actions or intentions | In Discussion, limitations section:  “*The intervention was selected, developed and implemented by the retailer, without the direct involvement of the research group. It is not possible to know the extent to which this was influenced by behavioural theory, prior commercial insights or awareness of government thinking, though it is probable that all contributed to greater or lesser extent.”* |
| Whilst a lot of information about the interventions and data used is provided in supplementary material (particularly in the combined paper), I would have liked to see this brought into the main text. The scant descriptions of the interventions on p6 of the combined paper left we many unanswered questions that I didn't feel were appropriate to be left to supplementary material. This includes exactly what products were impacted; how eg lower fat and low sugar are defined; whether products are added or moved from elsewhere in store etc. | We have tried to include all essential information in the methods section for the current study.  For the combined paper, we will deal with any changes in a separate re-submission. |  |
| The application of IMD and ethnicity to stores was poorly explained and, I felt, crude. Was this using lower level super output area of location? If so, how likely is it that supermarket customers are drawn only from the surrounding LSOA? At best, I think the limitations of this aspect of the work need further consideration. Perhaps it should be cast in an 'exploratory' light? | Unfortunately we have very limited information with regards to the ethnicity of the surrounding customer population. This is based on the retailer internal systems which classifies a store as having a predominantly White population of customers vs other ethnicities. For the store IMD, we used the postcode of the store to get information of the IMD decile where the store is located. We have added more information in the methods section about this, as well as an explanation in the discussion section on the limitations of the ethnicity classification provided by the retailer. | In Methods:  “*Store characteristics relating to the customer population included the English index of multiple deprivation (IMD) and ethnicity. The store postcode was matched to the IMD income domain, the official measure of relative deprivation in small areas in England [37]. The store sample covered neighbourhoods from deciles 1-10; re-grouped into IMD 1-3 (most deprived), 4-6 (mid) and 7-10 (least deprived). Ethnicity of the store customer population was classified by the retailer using internal proprietary systems, and grouped as predominantly white vs other-ethnicities*.”  In Discussion:  *“Finally, there was limited data on store characteristics, and the retailer provided only restricted data on the ethnicity of the customer population. The very broad categorisation of ethnicity is unlikely to have removed all of the confounding related to ethnicity in our results although there were no significant differences in the distribution of ethnicity between intervention and control stores.”* |
| I'd recommend both manuscripts are reviewed by a statistician. I wasn't clear what the hierarchical vs ITS analyses were contributing and how I should reconcile the information provided by both. More information on what specific questions these two different strategies addressed would be helpful. I found the inconsistent presentation of different information on the results for each intervention in the combined paper additionally confusing. Eg information on nutritional information and differences in pre-intervention sales are sometimes presented, sometimes not. | The analyses reported in both the confectionery Easter paper and the combined paper have followed the same approach reported in our previous paper (Piernas et al, Plos Medicine; <https://doi.org/10.1371/journal.pmed.1003715>) for which the statistical analysis plan was developed by a senior statistician.  Both the hierarchical model and the ITS model are useful to understand the impact of an intervention, but each approach covers different time periods. The hierarchical model is used to test average differences in sales between intervention and control stores over the entire period of time while the intervention is implemented (adjusting for baseline sales). The ITS is used to compare differences in sales between intervention vs control stores at the exact time of intervention implementation (referred as level change), and in the trend afterwards.  The information presented in both papers on store sales is consistent across all trials, and we have pre-intervention sales for all the studies showed here. However, the confectionery trial is the only one with available data on nutritional information of all sales, hence the reason for separating this trial from the rest. We have revised the manuscripts to make sure the information is consistent and have included more background on the use of both methods. |  |
| There were some apparent inconsistencies in the results that could do with more explanation and discussion. For example, in the ITS, I wasn't clear why there seemed to be substantial increases in the level for control stores at the point of intervention in many cases - even if not statistically significant, what do these reflect? I also wasn't clear if seasonal differences had been taken into account (both eg seasonal events such as Christmas, and seasonal patterns such as temperature changes in summer). This seems particularly important in the easter paper. Finally, the changes in some, but not all, nutrients of interest in line with sales (in the easter paper particularly, but I think in the combined paper too, but reporting of nutrients here was less consistent) could be explored further - there appears to be some displacement of purchases to other foods, but not total? | There are some trials where the control stores showed increased sales of the target products which is not surprising. This is particularly evident for the confectionery trial where both intervention and control stores showed increased sales of confectionery at the point of intervention, but this increase was less pronounced in the intervention stores, which happened because the intervention attenuated the seasonal increase in confectionery that would normally happen in the absence of intervention – the main finding of the paper.  With regards to seasonality, the most important consideration is whether seasonality appears to be similar between intervention and control stores. If so, then this should not be a major problem in the analyses.  As expected, the ITS models show weekly sales where it is evident that seasonal fluctuations appear to follow similar patterns in the intervention and control stores over the time period studied. Additionally, the difference in difference approach allows to further account for small time-related fluctuations and reduces the effect of absolute differences between the intervention and control stores in the estimations.  The reporting of changes in nutrients from total sales was only possible for the confectionery trial due to the lack of data for nutrients in total sales for the rest of the trials. |  |
| Overall the manuscripts need attention to terminology (and consistency) of this throughout - eg cost vs value; natural experiment vs natural experimental evaluation; interventions being short-lived vs intervention effects being short lived etc; the 'opposite' of the population approach is the high-risk approach, not the individual approach; some assertions are without reference (eg socioceoncomic inequalities exist); 'hypotheses' are referred to in the results, but none were presented in the aims. | We have revised our manuscript to ensure we have used consistent and accurate terminology |  |
|  |  |  |
| Reviewer #3 | Response | Changes |
| The authors present a nice study on reducing the availability of seasonal confectionary items from prominent grocery store locations (end of aisle) in an effort to curb over consumption of calorically dense foods. While the authors could not conduct a full RTC, they have baseline data from the prior year and a good number of stores designated as treatment vs. control in the intervention year.  Methods and Statistics: I found the stats to be a bit messy. The authors went through a pretty complicated procedure to try and match control and treatment grocery stores but there are some obvious limitations. First, even if they could be perfectly matched or randomly assigned, there are store-level differences that are inevitably unaccounted for. The authors acknowledge this, but there's still an element of preciseness to their results that seems unwarranted given the limitations of the sample and the data. It's also noteworthy that the intervention weakened over time -- even though the intervention intensified (pg 9) -- where the authors report that the initial difference between the treatment and control groups reduced over time. I don't believe the authors fully address this point in the discussion, but if I understand this correctly it has big implications for their findings. | We appreciate the reviewer positive comments.  With regards to the matching of the stores, one of the limitations of this retailer-led intervention is that the stores were not randomised. But we tried to investigate this further and we reported no differences in terms of the ethnicity of the customer population although there were differences in the IMD distribution of the stores due to the lack of stores in least deprived areas. Most importantly, we didn’t find significant differences in baseline sales between intervention and control stores.  Figure 1 shows the ITS analysis in which sales of confectionery almost overlap and show the exact same trend pre-intervention.  Therefore we are as confident as we can be that the selected stores were fairly comparable with regards to demographic characteristics, absolute sales and trends in sales pre-intervention. We cannot exclude other differences but we have no evidence of them.  We discussed the point about the waning of the intervention effect over time in the discussion. See below the next answer with regards to this. |  |
| Summary and conclusions: One question that lingers in any study where food/grocery options are limited by design is compensation (e.g., Schwartz, Riis, Elbel & Ariely 2012; Schwartz, Mochon, et al 2014). While it's great that the confectionary sales decreased in the intervention grocery stores, how do we know that shoppers didn't simply buy their Easter chocolate someplace else? The fact that the strength of the intervention weakened over time suggests that people will go out of their way to find these items when they aren't conveniently placed at the end of the aisle. Recent research also suggests this could be the case -- consumers in the Philadelphia area were shown to go to another county to buy SSB in order to avoid the soda tax. Denmark's fat tax had the unintended consequence of driving an increase in sales on dairy items in neighboring Germany. It would be nice to hear how the authors addressed this - did they follow up with shoppers to determine whether they purchased fewer confectionary items overall (regardless of where they shopped) compared to the prior year? I'm not sure how comprehensive the UK plan is for restricting access or promotions, but it seems that there's little incentive for grocery chains to cooperate with these promotional restrictions if they drive sales to competitors who don't cooperate. It's also possible that people will go deeper into the grocery store to find the items they want -- particularly around holidays when even disciplined shoppers are inclined to indulge. To that end, why would this intervention be better than some others, such as those that focus on reducing the portion sizes or sugar content? I would encourage the authors to think a bit beyond their initial results to determine the robustness of these findings and whether they are really going to achieve the policy goal of reducing confectionary consumption. I would also encourage them to scale back their claims -- fewer calories sold doesn't necessarily equate to fewer calories consumed if customers bought their confectionary items someplace else. | This trial showed that the seasonal increase in confectionery sales which would normally happen prior to Easter was attenuated after this intervention was implemented. That is, confectionary sales increased to a smaller extent than in previous periods (2018) or in control stores. This is evidenced by the results of the hierarchical model (Table 2) which showed significantly lower sales compared to 2018; as well as by the ITS models which show a significant difference in level at the time of intervention.  We were able to look at compensatory behaviours (within the same store) by looking at differences in the nutrient content of total sales (kcal, fat etc) which appear to also be significantly after the point of intervention. However we cannot exclude the possibility that people buy their confectionery in other retailers as we could only look at data from the participating retailer for this trial.  The fact that the intervention effect decreased over time is not surprising because this trial targeted seasonal confectionary products around Easter time (hence not around for the rest of the year). Once this period is over, especially towards the summer, it is normal to expect that both intervention and control stores reduce their sales of these kinds of confectionery. We do believe our results are robust in their aim to demonstrate a significant effect of this intervention which doesn’t seem to result in compensatory behaviours (at least within the same retailer) and this align with that the new policies are trying to achieve.  We have clarified these points in the discussion. | In Discussion:  “*For example, the duration of intervention was limited here because of the seasonal nature of the products, but future trials of positioning interventions should aim to try implement the intervention for a longer time period. This is important since the trends from ITS models after implementation suggested that the effect of the intervention may be short-lived, though this may be related to the seasonal nature of the products targeted (i.e. a large proportion of the products targeted in the intervention are not available the rest of the year).*  *Our analysis of the nutrient content of the total sales found no evidence of compensatory behaviours at least within the same retailer, although we cannot exclude the possibility that customers are purchasing confectionery in different stores where no restrictions are imposed*.” |
