# Supplementary material for: Removing seasonal confectionery from prominent store locations and purchasing behaviour within a major UK supermarket: Evaluation of a nonrandomised controlled intervention study
Source: PLoS Med. 2022 Mar 24;19(3):e1003951. doi: 10.1371/journal.pmed.1003951 (PMC8946674; doi:10.1371/journal.pmed.1003951)
Supplement: Attachment — Submitted filename: Response to reviewers CGF Conf trial 20 01 2022.docx [file pmed.1003951.s004.docx]

| Requests from the editors | | |
| --- | --- | --- |
| Comment | Response | Changes |
| Please revise your title to ‘Removing seasonal confectionery from prominent store locations within a major UK supermarket: Evaluation of a non-randomised controlled intervention study’. | We have revised the title accordingly | Title:  “*Removing seasonal confectionery from prominent store locations within a major UK supermarket: Evaluation of a non-randomised controlled intervention study*” |
| The final line in your Financial Disclosure (‘We thank the consumer goods…’) would be better placed in an Acknowledgements section at the end of the main text (as it does not pertain to funding received). | We have moved this section from the funding disclosure into the Acknowledgements section | Acknowledgements section |
| Line 23: Please refer to the study as a ‘non-randomised controlled intervention study’ rather than a non-randomised controlled trial (please check throughout the manuscript for consistency). | We have applied this change throughout the manuscript |  |
| Lines 45, 75, 278: Please avoid the term “effectiveness”. | We have removed this term throughout the manuscript and reworded the relevant sentences |  |
| Line 51: Please update to “… was associated with reductions in purchases …” or similar. | We have rephrased this sentence accordingly | Abstract, conclusions:  “*Removal of chocolate confectionery from prominent locations was associated with reduced purchases of these products, of sufficient magnitude to observe a reduction in the energy content of total food purchases.”* |
| Line 62: Update ‘governments’ to ‘the UK Government’. | We have updated this sentence to refer to the Government in England |  |
| Thank you for providing the STROBE checklist, please update to remove page numbers and use only section names and paragraph numbers (page numbers will change during revisions and may confuse the reader if inaccurate). | We have updated the STROBE checklist |  |
| line 51, suggest “… was associated with reductions in purchases …” or similar | See above |  |
| Line 252: Should this be ‘the level of weekly sales.’? | We have rephrased this sentence accordingly |  |
| Was there any attempt made to control for repeated measures in the data (i.e. the same individuals visiting the store multiple times during the study period)? | This analysis included store-level sales of the target product rather than individual purchasing data, hence we didn’t need to control for repeated measures within individuals. |  |
| Please provide the unadjusted comparisons as well as the adjusted comparisons in Table 2 (these can be presented separately in the supplementary materials if easier). | We have included unadjusted results in Table 2 | Table 2 |
| Comments from the reviewers: |  |  |
| Reviewer #1: Thanks authors for their great effort to improve the manuscript. I am satisfied with the response and revision. No further issues needing attention. | We thank the reviewer for their time and supportive comments |  |
| Reviewer #2: Thanks for responding to my previous comments. There is one issue that I still think deserve further attention. | We appreciate the reviewer time in reviewing this paper. |  |
| 1. IMD. I am still not clear at what geographical level IMD was calculated. Was is lower level super output area, middle level super output area, ward, local authority? All of these would involve linking postcode to another geography, but it is not clear from your ms which other geography you have used. I suspect you have used lower level super output area. The have an average population of just 1500 people. Hence the IMD of LSOA of location is likely to be a very poor marker of the deprivation of the people who shop in a store - particularly large out-of-town stores which most people drive to, and city center stores with high non-local usage. I still don't see this point discussed in consideration of the equity of effects seen. | The reviewer is right that the IMD used here reflects the relative deprivation at a small local area level (Lower-layer Super Output Areas) across England which covers an average of approximately 1,500 residents or 650 households. We appreciate this is a very crude measure of SES but it was the only appropriate one given the limited data provided to us by the supermarket (store postcode). We have clarified this in Methods and added a limitation regarding this. | Discussion, limitation section:  “*S*i*milarly, the IMD used as a measure of store deprivation may also be a very crude proxy for the SES status of the customer population, particularly when people drive to larger out-of-town supermarkets or for smaller stores located in city centres with a large proportion of non-local customers*” |
| Reviewer #3: Thanks to the authors for a thoughtful revision and set of comments on both papers. I have read the revised manuscripts, the revision notes, and my previous reviews. I remain positive about both papers, but many of my initial concerns remain. First, I don't think the authors can make the causal claims their titles or discussions of the results suggest. Other reviewers have also pointed this out. In my view, these papers are about seeing how various nudges are playing out in the field not about testing interventions. I equate this to non-experimental data that comes in after a drug that did well in clinical trials has been on the market for a while. You cannot overcome a lack of randomization with statistical massaging and/or a pre-post design. You cannot overcome underpowered studies with statistical massaging either. I don't think the word experiment should appear anywhere these papers. There is no natural experiment, as it would require some kind of exogenous shock that resulted in equivalent stores with equivalent populations be essentially randomized to treatment vs. control. I also have reservations about treating these studies as experimental given how little access the researchers had to interventions that were "selected, developed, and implemented" by the retailers -- who I assume are not experimentalists or data scientists. I understand the authors' desire to make a strong policy argument here and it's impressive to have so much data from several retailers trying to implement various nudges simultaneously. However, given the lack of data that's available for controls and robustness checks, I am very hesitant to sign off on a paper that makes causal claims about the viability of certain interventions. I think there are many interesting insights to be gleaned from the data but I think the authors should tone down their language that these results provide any sort of definitive test. I belabor this point because it's easy for keywords that appear in titles and abstracts to become sound bites for journalists and agenda-driven policy makers who never dig a little deeper into the work to realize that the claims cannot be substantiated by the data. | We thank the reviewer for their time and supportive comments.  In response to these comments and the editorial suggestions above, we have removed all instances referring to natural experiments, trials and effectiveness.  We have reworded the title to refer to this study as a non-randomised controlled intervention study |  |
