# Supplementary material for: Removing seasonal confectionery from prominent store locations and purchasing behaviour within a major UK supermarket: Evaluation of a nonrandomised controlled intervention study
Source: PLoS Med. 2022 Mar 24;19(3):e1003951. doi: 10.1371/journal.pmed.1003951 (PMC8946674; doi:10.1371/journal.pmed.1003951)
Supplement: Attachment — Submitted filename: Response to reviewers CGF Conf trial 17 02 2022.docx [file pmed.1003951.s005.docx]

| Requests from the editors | | |
| --- | --- | --- |
| Comment | Response | Changes |
| Please update the manuscript title to ‘Removing seasonal confectionery from prominent store locations and purchasing behaviour within a major UK supermarket: Evaluation of a non-randomised controlled intervention study’ (my apologies for omitting this suggestion in my previous revision letter). | We have changed the title accordingly | Title |
| In the Data Availability Statement, please provide further details as to how an interested researcher could establish contact with the retailer to request permission to access the data (this could be via an intermediary such as the University Research Office or the Institutional Review Board – please note that it cannot be via a study author). Feel free to contact me directly to discuss ([cdavidson@plos.org](mailto:cdavidson@plos.org)). | We have modified this statement to clarify how to establish contact with the retailer | This research was conducted according to a framework collaboration agreement  between the University of Oxford and the food retailers. Access to the study dataset by  external researchers is not permitted as this is defined as confidential information in the agreement. Access to the study data by external researchers will require the expressed written consent of the retailer. Please contact [hw@theconsumergoodsforum.com](mailto:hw@theconsumergoodsforum.com).  Access to the statistical code used in this analysis will be reviewed and granted upon request by the Nuffield Department of Primary Care PRimDISC committee (primdisc@phc.ox.ac.uk) |
| Line 55 – please remove the protocol registration details from the abstract. | Removed |  |
| Please remove subheadings from the Discussion section. | Removed |  |
| Please check all references for accuracy against our Submission Guidelines (<https://journals.plos.org/plosmedicine/s/submission-guidelines#loc-references>), in particular those for official reports (there are some issues with the authors listed for references 3, 10, 13, 14, 17, and references 18 and 47 are also not presented consistently). | We have revised all referenced to ensure consistency |  |
| For the panels in Figure 2, please either show the y-axes beginning at zero or show a break in the axes. | We have modified the plots in Figure 2 | Figure 2 |
| To help us extend the reach of your research, please provide any Twitter handle(s) that would be appropriate to tag, including your own, your coauthors’, your institution, funder, or lab. | [@OxPrimaryCare](http://www.twitter.com/oxprimarycare) |  |
