# Supplementary material for: Removing seasonal confectionery from prominent store locations and purchasing behaviour within a major UK supermarket: Evaluation of a nonrandomised controlled intervention study
Source: PLoS Med. 2022 Mar 24;19(3):e1003951. doi: 10.1371/journal.pmed.1003951 (PMC8946674; doi:10.1371/journal.pmed.1003951)
Supplement: Peer review history

# Editor report: Decision Letter 0

**DOI:** 10.1371/journal.pmed.1003951.r001

**Authors:** Callam Davidson

16 Sep 2021

Dear Dr Piernas, 

Thank you for submitting your manuscript entitled "A natural experiment testing removal of seasonal confectionery from prominent store locations: A non-randomised controlled trial in a major UK supermarket" for consideration by PLOS Medicine.

Your manuscript has now been evaluated by the PLOS Medicine editorial staff as well as by an academic editor with relevant expertise and I am writing to let you know that we would like to send your submission out for external peer review.

However, before we can send your manuscript to reviewers, we need you to complete your submission by providing the metadata that is required for full assessment. To this end, please login to Editorial Manager where you will find the paper in the 'Submissions Needing Revisions' folder on your homepage. Please click 'Revise Submission' from the Action Links and complete all additional questions in the submission questionnaire.

Please re-submit your manuscript within two working days, i.e. by Sep 20 2021 11:59PM.

Login to Editorial Manager here: https://www.editorialmanager.com/pmedicine

Once your full submission is complete, your paper will undergo a series of checks in preparation for peer review. Once your manuscript has passed all checks it will be sent out for review. 

Feel free to email us at plosmedicine@plos.org if you have any queries relating to your submission.

Kind regards,

Callam Davidson

Senior Editor

PLOS Medicine

# Peer review: Decision Letter 1

**DOI:** 10.1371/journal.pmed.1003951.r002

**Authors:** Callam Davidson

4 Nov 2021

Dear Dr. Piernas,

Thank you very much for submitting your manuscript "A natural experiment testing removal of seasonal confectionery from prominent store locations: A non-randomised controlled trial in a major UK supermarket" (PMEDICINE-D-21-03910R1) for consideration at PLOS Medicine. 

Your paper was evaluated by an associate editor and discussed among all the editors here. It was also discussed with an academic editor with relevant expertise, and sent to independent reviewers, including a statistical reviewer. The reviews are appended at the bottom of this email and any accompanying reviewer attachments can be seen via the link below:

[LINK]

In light of these reviews, I am afraid that we will not be able to accept the manuscript for publication in the journal in its current form, but we would like to consider a revised version that addresses the reviewers' and editors' comments. Obviously we cannot make any decision about publication until we have seen the revised manuscript and your response, and we plan to seek re-review by one or more of the reviewers. 

In revising the manuscript for further consideration, your revisions should address the specific points made by each reviewer and the editors. Please also check the guidelines for revised papers at http://journals.plos.org/plosmedicine/s/revising-your-manuscript for any that apply to your paper. In your rebuttal letter you should indicate your response to the reviewers' and editors' comments, the changes you have made in the manuscript, and include either an excerpt of the revised text or the location (eg: page and line number) where each change can be found. Please submit a clean version of the paper as the main article file; a version with changes marked should be uploaded as a marked up manuscript.

In addition, we request that you upload any figures associated with your paper as individual TIF or EPS files with 300dpi resolution at resubmission; please read our figure guidelines for more information on our requirements: http://journals.plos.org/plosmedicine/s/figures. While revising your submission, please upload your figure files to the PACE digital diagnostic tool, https://pacev2.apexcovantage.com/. PACE helps ensure that figures meet PLOS requirements. To use PACE, you must first register as a user. Then, login and navigate to the UPLOAD tab, where you will find detailed instructions on how to use the tool. If you encounter any issues or have any questions when using PACE, please email us at PLOSMedicine@plos.org.

We expect to receive your revised manuscript by Nov 25 2021 11:59PM. Please email us (plosmedicine@plos.org) if you have any questions or concerns.

***Please note while forming your response, if your article is accepted, you may have the opportunity to make the peer review history publicly available. The record will include editor decision letters (with reviews) and your responses to reviewer comments. If eligible, we will contact you to opt in or out.***

We ask every co-author listed on the manuscript to fill in a contributing author statement, making sure to declare all competing interests. If any of the co-authors have not filled in the statement, we will remind them to do so when the paper is revised. If all statements are not completed in a timely fashion this could hold up the re-review process. If new competing interests are declared later in the revision process, this may also hold up the submission. Should there be a problem getting one of your co-authors to fill in a statement we will be in contact. YOU MUST NOT ADD OR REMOVE AUTHORS UNLESS YOU HAVE ALERTED THE EDITOR HANDLING THE MANUSCRIPT TO THE CHANGE AND THEY SPECIFICALLY HAVE AGREED TO IT. You can see our competing interests policy here: http://journals.plos.org/plosmedicine/s/competing-interests.

Please use the following link to submit the revised manuscript: 

https://www.editorialmanager.com/pmedicine/

Your article can be found in the "Submissions Needing Revision" folder. 

To enhance the reproducibility of your results, we recommend that you deposit your laboratory protocols in protocols.io, where a protocol can be assigned its own identifier (DOI) such that it can be cited independently in the future. Additionally, PLOS ONE offers an option to publish peer-reviewed clinical study protocols. Read more information on sharing protocols at https://plos.org/protocols?utm_medium=editorial-email&utm_source=authorletters&utm_campaign=protocols

Please ensure that the paper adheres to the PLOS Data Availability Policy (see http://journals.plos.org/plosmedicine/s/data-availability), which requires that all data underlying the study's findings be provided in a repository or as Supporting Information. For data residing with a third party, authors are required to provide instructions with contact information for obtaining the data. PLOS journals do not allow statements supported by "data not shown" or "unpublished results." For such statements, authors must provide supporting data or cite public sources that include it. 

We look forward to receiving your revised manuscript. 

Sincerely,

Callam Davidson, 

PLOS Medicine

plosmedicine.org

-----------------------------------------------------------

Requests from the editors:

The editorial team would like to suggest combining the content of manuscript PMEDICINE-D-21-03910 and PMEDICINE-D-21-03912 into a single manuscript. You will receive a separate letter informing you that PMEDICINE-D-21-03912 has been rejected, however this is only to ensure the combined manuscript is processed as a single submission within Editorial Manager. The requests from the editors below, therefore, pertain to both of your submissions (henceforth referred to as 3910/3912).

Please use the reviewers’ comments to guide your structuring of the revised manuscript. It was generally felt that a more consistent and systematic presentation of the various interventions would facilitate interpretation of your findings. Reviewer 1 suggests that Supplementary tables 1 and 2 from the 3912 manuscript could be combined and presented as Table 1 in the main text to facilitate reader comprehension - this will also now require the inclusion of the study presented in 3910. 

Please revise your title according to reflect the combining of the two manuscripts. Your title must be nondeclarative and not a question. It should begin with main concept if possible. "Effect of" should be used only if causality can be inferred, i.e., for an RCT. Please place the study design ("A randomized controlled trial," "A retrospective study," "A modelling study," etc.) in the subtitle (ie, after a colon).

Please add a final line to your abstract ‘Methods and findings’ which summarises the main limitations (it should begin ‘The limitations of this study include’ or similar). Given that the manuscript covers a variety of studies, it may be best to focus on the limitations relating to study design more generally as opposed to detailing limitations specific to any one of the experiments presented. 

Please include line numbering in the margins throughout.

Citations should be in square brackets and occur before punctuation. 

As reflected in the reviewers’ reports, please provide further clarification as to the definition/classification of ethnicity in your studies and consider discussing it as a limitation.

The PDF conversion process appears to have introduced some minor formatting errors – an example can be found in line 1 of page 8 of 3910 and also line 3 of 3912 (a boxed question mark appears where it should not). Please check throughout for other errors. 

Please remove the funding information from the acknowledgements section (in the event of publication this will be published as metadata based on your responses to the submission form). 

Please also remove the Contributors, Competing interests, and Data sharing sections from the main text for the same reason as above. 

Please update your Data Availability Statement (in the submission form) to include your statement regarding availability of statistical code. Note that a study author cannot be the main contact for researchers wishing to obtain this code, it would be better if this can be shared in a public repository or handled by an alternative data management contact. Please see our policy here (https://journals.plos.org/plosmedicine/s/data-availability) and get in touch if you have any questions.

Please remove the Ethical approval statement from the end of the main text as this is already stated in the methods section.

In your references, please remove all formatting (italics) and only use et al. after listing the first six authors. Please also ensure you have provided the data accessed for any URLs provided. Our guidelines can be found here: https://journals.plos.org/plosmedicine/s/submission-guidelines#loc-references

Please ensure you define all abbreviations used in your tables (e.g. IMD, IRR). 

Thank you for providing a STROBE checklist. Please update the checklist to reflect the combined manuscript and please use section names and paragraph numbers rather than page numbers (these are likely to change during the revision process).

Please reference the checklist in your methods ("This study is reported as per the Strengthening the Reporting of Observational Studies in Epidemiology (STROBE) guideline (S1 Checklist)."

Thank you for providing an author summary, please update this to reflect the combined manuscript.

When reporting p-values, please use P<0.001 rather than P<0.0001 (please also check your figures and tables to ensure P<0.001 is used consistently). 

Comments from the reviewers:

Reviewer #1: This is a well-conducted non-randomised controlled trial testing removal of seasonal confectionery from prominent store locations in a major UK supermarket. The study design, sample size, datasets, statistical methods and analyses, and presentation (tables and figures) and interpretation of the results are mostly adequate and of a good standard. The key issue in the non-randomised controlled trial setting is to control the potential confounding factors and minimise the biases which the authors have done well by adequate store selection and matching, and also adjusting in the analyses for store demographic characteristics such as deprivation score IMD and ethnicity and average weekly sales over the baseline pre-intervention period. However, there are still a few relatively minor issues needing attention.

1) Adjustment for ethnicity. It's well known that London boroughs of Lambeth and Southwark have an urban multi-ethnic population with nearly half of population from ethnic minorities background, which is very different from other parts of the UK. In the paper, ethnicity is classified as predominantly white vs other ethnicities, which is a bit simplistic and non-specific. It is likely to have residual confounding in ethnicity even after such adjustment in the paper. It would be good if the authors can discuss this as a limitation in the paper.

2) In the abstract, it says "ITS analyses showed statistically significant differences in the level at the point of intervention (P<0·05 for confectionery sales)". As the authors are using 3 digits for P-values in the paper, it would be good to be consistent throughout. I am aware here P<0.05 refers to a range of p-values but can replace with, for example, P ranges from 0.024 to 0.064. Also, in the next sentence "but also in the trend afterwards, indicating that the initial difference between intervention and control stores reduced over time", p-value is needed to support the statement.

3) In the first paragraph in the discussion, it says "This intervention to remove chocolate confectionery in prominent areas of the store attenuated the seasonal increase in confectionery sales, with an overall absolute difference between intervention and control stores of approximately 127 units (~21 kg) of confectionery per store per week". However, this 127 units just appeared out of blue and I had to figure it out myself. It's not mentioned in table or the results section at all, and it's not the primary outcome either. It would be good to give a message consistent with what's in the abtract which is on percentage differences in sales and etc. The absolute difference can be described as secondary but can't replace the primary outcome.

Reviewer #2: I was asked to review these papers together. Given their similarity many of my comments apply equally to both and so I have provided a combined report. I've noted where comments refer particularly to one or the other paper and described these as the 'combined' or 'easter' papers. Overall, I wasn't convinced by the value of separating these papers. Whilst the combined paper is certainly complicated, I don't think that adding the easter analyses would make it appreciably more so. I think perhaps the only value of the distinct easter paper is that it speaks very specifically to current UK public health policy decisions. On the other hand, the specific high value placement message could be drawn out in post-publication dissemination and, arguably, UK public health policy needs to be thinking beyond just the current policy decisions on the table to some of the strategies presented in the combined paper.

These papers report on a rather disparate set of interventions in supermarkets aiming to improve the healthiness of purchases. I applaud the authors on the partnership they have developed with the respective retailers and agree that this represents a substantial achievement both in terms of interventions achieved and in data made available. Unfortunately I found the report (particularly in the combined paper) of, what is undoubtedly a complicated collection of work, rather hard to follow and a little underdeveloped in terms of both theoretical introduction and interpretation of findings. 

The nature of the partnership between the researchers and the retailers is a little unclear. Although there are a number of different definitions of natural experiments, and natural experimental evaluations, key is that the nature and implementation of the intervention is outwith control of the researchers. It is not clear if this was the case here. If there is uncertainty in definitions, non-randomised controlled trials is an unambiguous description of what was done.

I'd also like to understand more about why this particular group of interventions was selected for study. Was this informed by theory, pragmatic considerations or something else? A key limitation on how the findings can be interpreted is the possibility of an intervention modality by food category interaction - as different food categories were selected for each intervention modality, we can't tell if the effects reported are specific to the food category, the intervention modality, or the interaction of the two. I'd like to see more consideration given to this throughout.

I also thought there was a missed opportunity to raise the issue of industry versus government led public health initiatives in this paper. Again, it wasn't clear to me whether the decision to implement these interventions was driven by retailers, the industry body mentioned, the charity mentioned, or the researchers. Shedding some further light on this may help illuminate how such interventions might be implemented more consistently.

Whilst a lot of information about the interventions and data used is provided in supplementary material (particularly in the combined paper), I would have liked to see this brought into the main text. The scant descriptions of the interventions on p6 of the combined paper left we many unanswered questions that I didn't feel were appropriate to be left to supplementary material. This includes exactly what products were impacted; how eg lower fat and low sugar are defined; whether products are added or moved from elsewhere in store etc.

The application of IMD and ethnicity to stores was poorly explained and, I felt, crude. Was this using lower level super output area of location? If so, how likely is it that supermarket customers are drawn only from the surrounding LSOA? At best, I think the limitations of this aspect of the work need further consideration. Perhaps it should be cast in an 'exploratory' light?

I'd recommend both manuscripts are reviewed by a statistician. I wasn't clear what the hierarchical vs ITS analyses were contributing and how I should reconcile the information provided by both. More information on what specific questions these two different strategies addressed would be helpful. I found the inconsistent presentation of different information on the results for each intervention in the combined paper additionally confusing. Eg information on nutritional information and differences in pre-intervention sales are sometimes presented, sometimes not. 

There were some apparent inconsistencies in the results that could do with more explanation and discussion. For example, in the ITS, I wasn't clear why there seemed to be substantial increases in the level for control stores at the point of intervention in many cases - even if not statistically significant, what do these reflect? I also wasn't clear if seasonal differences had been taken into account (both eg seasonal events such as Christmas, and seasonal patterns such as temperature changes in summer). This seems particularly important in the easter paper. Finally, the changes in some, but not all, nutrients of interest in line with sales (in the easter paper particularly, but I think in the combined paper too, but reporting of nutrients here was less consistent) could be explored further - there appears to be some displacement of purchases to other foods, but not total?

I would like to see more consideration in the discussion section (of the combined paper) of why some intervention modalities seemed more promising than others. The authors present a general background of inconsistent previous literature. What do these findings add? Are we any further forward in working out in what contexts different intervention modalities may be more or less effective? How could we make progress on this?

Overall the manuscripts need attention to terminology (and consistency) of this throughout - eg cost vs value; natural experiment vs natural experimental evaluation; interventions being short-lived vs intervention effects being short lived etc; the 'opposite' of the population approach is the high-risk approach, not the individual approach; some assertions are without reference (eg socioceoncomic inequalities exist); 'hypotheses' are referred to in the results, but none were presented in the aims.

In relation to the consideration of Adams et al (2016), please be clearer that the hypothesis concerning 'agency' is proposed, not stated; and that the agentic effort referred to is specifically of individual potential beneficiaries of interventions - interventions don't have agency, they require individual recipients to use their agency and mobilise their resources in order to benefit.

Reviewer #3: The authors present a nice study on reducing the availability of seasonal confectionary items from prominent grocery store locations (end of aisle) in an effort to curb over consumption of calorically dense foods. While the authors could not conduct a full RTC, they have baseline data from the prior year and a good number of stores designated as treatment vs. control in the intervention year. 

Methods and Statistics: I found the stats to be a bit messy. The authors went through a pretty complicated procedure to try and match control and treatment grocery stores but there are some obvious limitations. First, even if they could be perfectly matched or randomly assigned, there are store-level differences that are inevitably unaccounted for. The authors acknowledge this, but there's still an element of preciseness to their results that seems unwarranted given the limitations of the sample and the data. It's also noteworthy that the intervention weakened over time -- even though the intervention intensified (pg 9) -- where the authors report that the initial difference between the treatment and control groups reduced over time. I don't believe the authors fully address this point in the discussion, but if I understand this correctly it has big implications for their findings. 

Summary and conclusions: One question that lingers in any study where food/grocery options are limited by design is compensation (e.g., Schwartz, Riis, Elbel & Ariely 2012; Schwartz, Mochon, et al 2014). While it's great that the confectionary sales decreased in the intervention grocery stores, how do we know that shoppers didn't simply buy their Easter chocolate someplace else? The fact that the strength of the intervention weakened over time suggests that people will go out of their way to find these items when they aren't conveniently placed at the end of the aisle. Recent research also suggests this could be the case -- consumers in the Philadelphia area were shown to go to another county to buy SSB in order to avoid the soda tax. Denmark's fat tax had the unintended consequence of driving an increase in sales on dairy items in neighboring Germany. It would be nice to hear how the authors addressed this - did they follow up with shoppers to determine whether they purchased fewer confectionary items overall (regardless of where they shopped) compared to the prior year? I'm not sure how comprehensive the UK plan is for restricting access or promotions, but it seems that there's little incentive for grocery chains to cooperate with these promotional restrictions if they drive sales to competitors who don't cooperate. It's also possible that people will go deeper into the grocery store to find the items they want -- particularly around holidays when even disciplined shoppers are inclined to indulge. To that end, why would this intervention be better than some others, such as those that focus on reducing the portion sizes or sugar content? I would encourage the authors to think a bit beyond their initial results to determine the robustness of these findings and whether they are really going to achieve the policy goal of reducing confectionary consumption. I would also encourage them to scale back their claims -- fewer calories sold doesn't necessarily equate to fewer calories consumed if customers bought their confectionary items someplace else.

Any attachments provided with reviews can be seen via the following link:

[LINK]

# Author comment: Author response to Decision Letter 1

**DOI:** 10.1371/journal.pmed.1003951.r003

5 Dec 2021

Attachment Submitted filename: Response to reviewers CGF Conf trial 02 12 2021.docx Click here for additional data file.

# Peer review: Decision Letter 2

**DOI:** 10.1371/journal.pmed.1003951.r004

**Authors:** Callam Davidson

17 Jan 2022

Dear Dr. Piernas,

Thank you very much for submitting your revised manuscript "A natural experiment removing seasonal confectionery from prominent store locations within in a major UK supermarket: A non-randomised controlled trial" (PMEDICINE-D-21-03910R2) for consideration at PLOS Medicine. 

Your paper was evaluated by an associate editor and discussed among all the editors here. It was also discussed with an academic editor with relevant expertise, and sent back to the reviewers. The reviews are appended at the bottom of this email and any accompanying reviewer attachments can be seen via the link below:

[LINK]

In light of these reviews, we will not be able to accept the manuscript for publication in the journal in its current form, but we would like to consider a revised version that addresses the reviewers' and editors' comments. We cannot make any decision about publication until we have seen the revised manuscript and your response, and we plan to seek re-review by one or more of the reviewers. 

In revising the manuscript for further consideration, your revisions should address the specific points made by each reviewer and the editors. Please also check the guidelines for revised papers at http://journals.plos.org/plosmedicine/s/revising-your-manuscript for any that apply to your paper. In your rebuttal letter you should indicate your response to the reviewers' and editors' comments, the changes you have made in the manuscript, and include either an excerpt of the revised text or the location (eg: page and line number) where each change can be found. Please submit a clean version of the paper as the main article file; a version with changes marked should be uploaded as a marked up manuscript.

In addition, we request that you upload any figures associated with your paper as individual TIF or EPS files with 300dpi resolution at resubmission; please read our figure guidelines for more information on our requirements: http://journals.plos.org/plosmedicine/s/figures. While revising your submission, please upload your figure files to the PACE digital diagnostic tool, https://pacev2.apexcovantage.com/. PACE helps ensure that figures meet PLOS requirements. To use PACE, you must first register as a user. Then, login and navigate to the UPLOAD tab, where you will find detailed instructions on how to use the tool. If you encounter any issues or have any questions when using PACE, please email us at PLOSMedicine@plos.org.

We hope to receive your revised manuscript by Feb 07 2022 11:59PM. Please email us (plosmedicine@plos.org) if you have any questions or concerns.

***Please note while forming your response, if your article is accepted, you may have the opportunity to make the peer review history publicly available. The record will include editor decision letters (with reviews) and your responses to reviewer comments. If eligible, we will contact you to opt in or out.***

We ask every co-author listed on the manuscript to fill in a contributing author statement, making sure to declare all competing interests. If any of the co-authors have not filled in the statement, we will remind them to do so when the paper is revised. If all statements are not completed in a timely fashion this could hold up the re-review process. If new competing interests are declared later in the revision process, this may also hold up the submission. Should there be a problem getting one of your co-authors to fill in a statement we will be in contact. YOU MUST NOT ADD OR REMOVE AUTHORS UNLESS YOU HAVE ALERTED THE EDITOR HANDLING THE MANUSCRIPT TO THE CHANGE AND THEY SPECIFICALLY HAVE AGREED TO IT. You can see our competing interests policy here: http://journals.plos.org/plosmedicine/s/competing-interests.

Please use the following link to submit the revised manuscript: 

https://www.editorialmanager.com/pmedicine/

Your article can be found in the "Submissions Needing Revision" folder. 

To enhance the reproducibility of your results, we recommend that you deposit your laboratory protocols in protocols.io, where a protocol can be assigned its own identifier (DOI) such that it can be cited independently in the future. Additionally, PLOS ONE offers an option to publish peer-reviewed clinical study protocols. Read more information on sharing protocols at https://plos.org/protocols?utm_medium=editorial-email&utm_source=authorletters&utm_campaign=protocols

Please ensure that the paper adheres to the PLOS Data Availability Policy (see http://journals.plos.org/plosmedicine/s/data-availability), which requires that all data underlying the study's findings be provided in a repository or as Supporting Information. For data residing with a third party, authors are required to provide instructions with contact information for obtaining the data. PLOS journals do not allow statements supported by "data not shown" or "unpublished results." For such statements, authors must provide supporting data or cite public sources that include it. 

We look forward to receiving your revised manuscript. 

Sincerely,

Callam Davidson, 

Associate Editor

PLOS Medicine

plosmedicine.org

-----------------------------------------------------------

Requests from the editors:

Please revise your title to ‘Removing seasonal confectionery from prominent store locations within a major UK supermarket: Evaluation of a non-randomised controlled intervention study’.

The final line in your Financial Disclosure (‘We thank the consumer goods…’) would be better placed in an Acknowledgements section at the end of the main text (as it does not pertain to funding received).

Line 23: Please refer to the study as a ‘non-randomised controlled intervention study’ rather than a non-randomised controlled trial (please check throughout the manuscript for consistency).

Lines 45, 75, 278: Please avoid the term “effectiveness”.

Line 51: Please update to “… was associated with reductions in purchases …” or similar.

Line 62: Update ‘governments’ to ‘the UK Government’.

Thank you for providing the STROBE checklist, please update to remove page numbers and use only section names and paragraph numbers (page numbers will change during revisions and may confuse the reader if inaccurate). 

 line 51, suggest “… was associated with reductions in purchases …” or similar

Line 252: Should this be ‘the level of weekly sales.’?

Was there any attempt made to control for repeated measures in the data (i.e. the same individuals visiting the store multiple times during the study period)?

Please provide the unadjusted comparisons as well as the adjusted comparisons in Table 2 (these can be presented separately in the supplementary materials if easier). 

Comments from the reviewers:

Reviewer #1: Thanks authors for their great effort to improve the manuscript. I am satisfied with the response and revision. No further issues needing attention.

Reviewer #2: Thanks for responding to my previous comments. There is one issue that I still think deserve further attention.

1. IMD. I am still not clear at what geographical level IMD was calculated. Was is lower level super output area, middle level super output area, ward, local authority? All of these would involve linking postcode to another geography, but it is not clear from your ms which other geography you have used. I suspect you have used lower level super output area. The have an average population of just 1500 people. Hence the IMD of LSOA of location is likely to be a very poor marker of the deprivation of the people who shop in a store - particularly large out-of-town stores which most people drive to, and city center stores with high non-local usage. I still don't see this point discussed in consideration of the equity of effects seen.

Reviewer #3: Thanks to the authors for a thoughtful revision and set of comments on both papers. I have read the revised manuscripts, the revision notes, and my previous reviews. I remain positive about both papers, but many of my initial concerns remain. First, I don't think the authors can make the causal claims their titles or discussions of the results suggest. Other reviewers have also pointed this out. In my view, these papers are about seeing how various nudges are playing out in the field not about testing interventions. I equate this to non-experimental data that comes in after a drug that did well in clinical trials has been on the market for a while. You cannot overcome a lack of randomization with statistical massaging and/or a pre-post design. You cannot overcome underpowered studies with statistical massaging either. I don't think the word experiment should appear anywhere these papers. There is no natural experiment, as it would require some kind of exogenous shock that resulted in equivalent stores with equivalent populations be essentially randomized to treatment vs. control. I also have reservations about treating these studies as experimental given how little access the researchers had to interventions that were "selected, developed, and implemented" by the retailers -- who I assume are not experimentalists or data scientists. I understand the authors' desire to make a strong policy argument here and it's impressive to have so much data from several retailers trying to implement various nudges simultaneously. However, given the lack of data that's available for controls and robustness checks, I am very hesitant to sign off on a paper that makes causal claims about the viability of certain interventions. I think there are many interesting insights to be gleaned from the data but I think the authors should tone down their language that these results provide any sort of definitive test. I belabor this point because it's easy for keywords that appear in titles and abstracts to become sound bites for journalists and agenda-driven policy makers who never dig a little deeper into the work to realize that the claims cannot be substantiated by the data.

Any attachments provided with reviews can be seen via the following link:

[LINK]

# Author comment: Author response to Decision Letter 2

**DOI:** 10.1371/journal.pmed.1003951.r005

20 Jan 2022

Attachment Submitted filename: Response to reviewers CGF Conf trial 20 01 2022.docx Click here for additional data file.

# Peer review: Decision Letter 3

**DOI:** 10.1371/journal.pmed.1003951.r006

**Authors:** Callam Davidson

15 Feb 2022

Dear Dr. Piernas,

Thank you very much for re-submitting your manuscript "Removing seasonal confectionery from prominent store locations within a major UK supermarket: Evaluation of a non-randomised controlled intervention study" (PMEDICINE-D-21-03910R3) for review by PLOS Medicine.

I have discussed the paper with my colleagues and the academic editor and it was also seen again by two reviewers. I am pleased to say that provided the remaining editorial and production issues are dealt with we are planning to accept the paper for publication in the journal.

The remaining issues that need to be addressed are listed at the end of this email. Any accompanying reviewer attachments can be seen via the link below. Please take these into account before resubmitting your manuscript:

[LINK]

***Please note while forming your response, if your article is accepted, you may have the opportunity to make the peer review history publicly available. The record will include editor decision letters (with reviews) and your responses to reviewer comments. If eligible, we will contact you to opt in or out.***

In revising the manuscript for further consideration here, please ensure you address the specific points made by each reviewer and the editors. In your rebuttal letter you should indicate your response to the reviewers' and editors' comments and the changes you have made in the manuscript. Please submit a clean version of the paper as the main article file. A version with changes marked must also be uploaded as a marked up manuscript file.

Please also check the guidelines for revised papers at http://journals.plos.org/plosmedicine/s/revising-your-manuscript for any that apply to your paper. If you haven't already, we ask that you provide a short, non-technical Author Summary of your research to make findings accessible to a wide audience that includes both scientists and non-scientists. The Author Summary should immediately follow the Abstract in your revised manuscript. This text is subject to editorial change and should be distinct from the scientific abstract.

We hope to receive your revised manuscript within 1 week. Please email us (plosmedicine@plos.org) if you have any questions or concerns.

We ask every co-author listed on the manuscript to fill in a contributing author statement. If any of the co-authors have not filled in the statement, we will remind them to do so when the paper is revised. If all statements are not completed in a timely fashion this could hold up the re-review process. Should there be a problem getting one of your co-authors to fill in a statement we will be in contact. YOU MUST NOT ADD OR REMOVE AUTHORS UNLESS YOU HAVE ALERTED THE EDITOR HANDLING THE MANUSCRIPT TO THE CHANGE AND THEY SPECIFICALLY HAVE AGREED TO IT.

Please ensure that the paper adheres to the PLOS Data Availability Policy (see http://journals.plos.org/plosmedicine/s/data-availability), which requires that all data underlying the study's findings be provided in a repository or as Supporting Information. For data residing with a third party, authors are required to provide instructions with contact information for obtaining the data. PLOS journals do not allow statements supported by "data not shown" or "unpublished results." For such statements, authors must provide supporting data or cite public sources that include it.

To enhance the reproducibility of your results, we recommend that you deposit your laboratory protocols in protocols.io, where a protocol can be assigned its own identifier (DOI) such that it can be cited independently in the future. Additionally, PLOS ONE offers an option to publish peer-reviewed clinical study protocols. Read more information on sharing protocols at https://plos.org/protocols?utm_medium=editorial-email&utm_source=authorletters&utm_campaign=protocols

Please review your reference list to ensure that it is complete and correct. If you have cited papers that have been retracted, please include the rationale for doing so in the manuscript text, or remove these references and replace them with relevant current references. Any changes to the reference list should be mentioned in the rebuttal letter that accompanies your revised manuscript.

Please note, when your manuscript is accepted, an uncorrected proof of your manuscript will be published online ahead of the final version, unless you've already opted out via the online submission form. If, for any reason, you do not want an earlier version of your manuscript published online or are unsure if you have already indicated as such, please let the journal staff know immediately at plosmedicine@plos.org.

If you have any questions in the meantime, please contact me or the journal staff on plosmedicine@plos.org.  

We look forward to receiving the revised manuscript by Feb 22 2022 11:59PM.   

Sincerely,

Callam Davidson, 

Associate Editor 

PLOS Medicine

plosmedicine.org

------------------------------------------------------------

Requests from Editors:

Please update the manuscript title to ‘Removing seasonal confectionery from prominent store locations and purchasing behaviour within a major UK supermarket: Evaluation of a non-randomised controlled intervention study’ (my apologies for omitting this suggestion in my previous revision letter). 

In the Data Availability Statement, please provide further details as to how an interested researcher could establish contact with the retailer to request permission to access the data (this could be via an intermediary such as the University Research Office or the Institutional Review Board – please note that it cannot be via a study author). Feel free to contact me directly to discuss (cdavidson@plos.org).

Line 55 – please remove the protocol registration details from the abstract.

Please remove subheadings from the Discussion section. 

Please check all references for accuracy against our Submission Guidelines (https://journals.plos.org/plosmedicine/s/submission-guidelines#loc-references), in particular those for official reports (there are some issues with the authors listed for references 3, 10, 13, 14, 17, and references 18 and 47 are also not presented consistently).

For the panels in Figure 2, please either show the y-axes beginning at zero or show a break in the axes.

To help us extend the reach of your research, please provide any Twitter handle(s) that would be appropriate to tag, including your own, your coauthors’, your institution, funder, or lab.

Comments from Reviewers:

Reviewer #2: Thanks for responding to my remaining comment. I've not further comments. Congratulations on some great work.

Reviewer #3: Thanks for being responsive to all the comments and questions. Nice work!

Any attachments provided with reviews can be seen via the following link:

[LINK]

# Author comment: Author response to Decision Letter 3

**DOI:** 10.1371/journal.pmed.1003951.r007

17 Feb 2022

Attachment Submitted filename: Response to reviewers CGF Conf trial 17 02 2022.docx Click here for additional data file.

# Editor report: Decision Letter 4

**DOI:** 10.1371/journal.pmed.1003951.r008

**Authors:** Callam Davidson

23 Feb 2022

Dear Dr Piernas, 

On behalf of my colleagues and the Academic Editor, Dr Jean Adams, I am pleased to inform you that we have agreed to publish your manuscript "Removing seasonal confectionery from prominent store locations and purchasing behaviour within a major UK supermarket: Evaluation of a non-randomised controlled intervention study" (PMEDICINE-D-21-03910R4) in PLOS Medicine.

Before your manuscript can be formally accepted you will need to complete some formatting changes, which you will receive in a follow up email. Please be aware that it may take several days for you to receive this email; during this time no action is required by you. Once you have received these formatting requests, please note that your manuscript will not be scheduled for publication until you have made the required changes.

When making the formatting changes, please also review your references to ensure they meet our guidelines (https://journals.plos.org/plosmedicine/s/submission-guidelines#loc-references). Journal name abbreviations should be those found in the National Center for Biotechnology Information (NCBI) databases (https://www.ncbi.nlm.nih.gov/nlmcatalog/journals). 

In the meantime, please log into Editorial Manager at http://www.editorialmanager.com/pmedicine/, click the "Update My Information" link at the top of the page, and update your user information to ensure an efficient production process. 

PRESS

We frequently collaborate with press offices. If your institution or institutions have a press office, please notify them about your upcoming paper at this point, to enable them to help maximise its impact. If the press office is planning to promote your findings, we would be grateful if they could coordinate with medicinepress@plos.org. If you have not yet opted out of the early version process, we ask that you notify us immediately of any press plans so that we may do so on your behalf.

We also ask that you take this opportunity to read our Embargo Policy regarding the discussion, promotion and media coverage of work that is yet to be published by PLOS. As your manuscript is not yet published, it is bound by the conditions of our Embargo Policy. Please be aware that this policy is in place both to ensure that any press coverage of your article is fully substantiated and to provide a direct link between such coverage and the published work. For full details of our Embargo Policy, please visit http://www.plos.org/about/media-inquiries/embargo-policy/.

To enhance the reproducibility of your results, we recommend that you deposit your laboratory protocols in protocols.io, where a protocol can be assigned its own identifier (DOI) such that it can be cited independently in the future. Additionally, PLOS ONE offers an option to publish peer-reviewed clinical study protocols. Read more information on sharing protocols at https://plos.org/protocols?utm_medium=editorial-email&utm_source=authorletters&utm_campaign=protocols

Thank you again for submitting to PLOS Medicine. We look forward to publishing your paper. 

Sincerely, 

Callam Davidson 

Associate Editor 

PLOS Medicine
